# Discovery of the Streamlined Haloarchaeon *Halorutilus salinus*, Comprising a New Order Widespread in Hypersaline Environments across the World

**DOI:** 10.1128/msystems.01198-22

**Published:** 2023-03-21

**Authors:** Ana Durán-Viseras, Cristina Sánchez-Porro, Tomeu Viver, Konstantinos T. Konstantinidis, Antonio Ventosa

**Affiliations:** a Department of Microbiology and Parasitology, Faculty of Pharmacy, University of Sevilla, Sevilla, Spain; b School of Civil & Environmental Engineering, Georgia Institute of Technology, Atlanta, Georgia, USA; c Marine Microbiology Group, Department of Animal and Microbial Biodiversity, Mediterranean Institute for Advanced Studies (IMEDEA, CSIC-UIB), Esporles, Spain; Ocean University of China

**Keywords:** hypersaline environments, *Halorutilales* ord. nov., comparative genomics, streamlining, RuBisCO, microbial ecology, osmoadaptation

## Abstract

The class *Halobacteria* is one of the most diverse groups within the *Euryarchaeota* phylum, whose members are ubiquitously distributed in hypersaline environments, where they often constitute the major population. Here, we report the discovery and isolation of a new halophilic archaeon, strain F3-133^T^ exhibiting ≤86.3% 16S rRNA gene identity to any previously cultivated archaeon, and, thus, representing a new order. Analysis of available 16S rRNA gene amplicon and metagenomic data sets showed that the new isolate represents an abundant group in intermediate-to-high salinity ecosystems and is widely distributed across the world. The isolate presents a streamlined genome, which probably accounts for its ecological success in nature and its fastidious growth in culture. The predominant osmoprotection mechanism appears to be the typical *salt-in* strategy used by other haloarchaea. Furthermore, the genome contains the complete gene set for nucleotide monophosphate degradation pathway through archaeal RuBisCO, being within the first halophilic archaea representatives reported to code this enzyme. Genomic comparisons with previously described representatives of the phylum *Euryarchaeota* were consistent with the 16S rRNA gene data in supporting that our isolate represents a novel order within the class *Halobacteria* for which we propose the names *Halorutilales* ord. nov., *Halorutilaceae* fam. nov., *Halorutilus* gen. nov. and *Halorutilus salinus* sp. nov.

**IMPORTANCE** The discovery of the new halophilic archaeon, *Halorutilus salinus*, representing a novel order, family, genus, and species within the class *Halobacteria* and phylum *Euryarchaeota* clearly enables insights into the microbial dark matter, expanding the current taxonomical knowledge of this group of archaea. The in-depth comparative genomic analysis performed on this new taxon revealed one of the first known examples of an *Halobacteria* representative coding the archaeal RuBisCO gene and with a streamlined genome, being ecologically successful in nature and explaining its previous non-isolation. Altogether, this research brings light into the understanding of the physiology of the *Halobacteria* class members, their ecological distribution, and capacity to thrive in hypersaline environments.

## INTRODUCTION

Hypersaline ecosystems are extreme environments characterized by high salt concentrations, much greater than that of seawater and often close to salt saturation (1, 2). The interest of the scientific community on hypersaline environments have been hugely increased as possible analogues to life on other planets such as Mars (3–5), as well as by its biotechnological potential in the biomedicine and biotechnology fields (6–8).

These ecosystems, whether natural or manmade, are characterized by severe stresses for microbial organisms, due not only to high salt concentration, but to other factors, such as low nutrient and oxygen availability, alkalinity, high UV irradiance, and the possible presence of toxic compounds and heavy metals (2, 9). These conditions have selected for low class/family diversity, generally consisting of two major lineages i.e., the archaeal *Halobacteria* class and the bacterial family of *Salinibacteraceae*, class *Rhodothermia* (10, 11), but with relatively high species richness (microdiversity) within each class (12, 13). Members of the class *Halobacteria* are among the best adapted organisms to deal with the high salt concentration encountered in those habitats (14, 15), and are widely distributed in hypersaline systems, from marine salterns to salt lakes, coastal sabkhas, salt mines, hypersaline soda lakes, hypersaline soils, and salt-fermented seafood (15–17).

The class *Halobacteria* of the phylum *Euryarchaeota* is one of the most diverse groups within the domain *Archaea* that, at the time of this writing, is constituted by 71 genera and 308 species (18, 19). Currently, the class *Halobacteria* is divided into 3 orders (*Halobacteriales*, *Haloferacales*, and *Natrialbales*), that each comprises 3 (*Haloarculaceae*, *Halobacteriaceae*, and *Halococcaceae*), 2 (*Haloferacaceae* and *Halorubraceae*) and 1 (*Natrialbaceae*) families, respectively (7, 20, 21).

Although traditional cultivation techniques have led to the isolation and description of hundreds of new groups of halophilic bacteria and archaea (19, 22–30), a great number of recent culture-independent studies from hypersaline habitats have revealed the prominence of several yet-uncultivated lineages in these systems (31–34). Most of these uncultivated groups represent dominant populations in hypersaline environments (31, 32, 35, 36), and are presumably slow growing and fastidious to isolate microorganisms. Thus, persistent efforts and improvements of the cultivation methodologies are still needed to bring these uncultivated lineages to culture (37). Within this context the term “culturomics” was coined to denote the application of high-throughput culturing efforts for the identification of a higher microbial culturable diversity (38).

Our recent exploration of the microbial diversity in Isla Cristina salterns (Huelva, Spain) following the “culturomics” approach (39, 40), has yielded the isolation of several novel haloarchaeal taxa, such as the new genera *Halosegnis* (40) and *Haloglomus* (26), as well as several new species of the genera *Halonotius* (41, 42) and *Natronomonas* (43). Indeed, subsequent ecological studies highlighted the wide geographical distribution of these groups and their substantial abundance in different hypersaline systems (26, 40, 42, 44–47).

As part of the same isolation campaign in the multi-pond, manmade Isla Cristina saltern, a novel haloarchaeon, designated as strain F3-133^T^, was isolated in pure culture. Based on the genome-aggregate average amino acid identity (AAI; ≤ 51.7%) and 16S rRNA gene (≤ 86.3% identity), strain F3-133^T^ was shown to be distantly related to any previously described haloarchaea. Here, we report the characteristic of its genome sequence, its environmental distribution based on available 16S rRNA gene amplicon and metagenomic data sets, as well as the ecophysiological strategies that underly its ecological success.

## RESULTS AND DISCUSSION

### Isolation of strain F3-133^T^ by culturomics.

The culturomics approach was applied with the aim of isolating and characterizing the functional roles of the abundant saltern taxa lacking currently cultured representatives. Based on previously successful methodological approaches, for the isolation of other elusive microorganisms (26, 40), several growth media and conditions were tested. The water samples collected from the different saltern ponds of Isla Cristina (37° 12′ N 7° 19′ W) were serially diluted and plated on the different culture media. Plates were subsequently incubated under different conditions (aerobic, microaerophilic, or anaerobic, in dark or light) and for long-time periods (up to 3 months). During this period, colonies appearing on plates were carefully observed, and those of fastest growth and large morphology were labeled in order to be further discarded. Afterwards, an arduous high-throughput isolation screening of those unlabeled colonies with slow growth and tiny morphology was conducted resulting in more than 2,000 newly-isolated-single-colonies. These isolates were identified by 16S rRNA gene amplification. Isolates likely to represent new groups of microorganisms based on 16S rRNA gene identity, were selected for further characterization. Among them, 1 tiny, red-pigmented colony (<0.5 mm) with extremely slow growth, designated strain F3-133^T^, exhibited a remarkably low relatedness with any previously cultivated haloarchaea. Strain F3-133^T^ was isolated from a culture medium containing pyruvate, which indeed has been considered as a key compound for the isolation of fastidious microorganisms (48), such as the halophilic archaeon *Haloquadratum waslbyi*, or the streamlined halophilic and marine bacteria Spiribacter salinus and *Pelagibacter ubique*, among others. Due to its very slow and limited growth under laboratory conditions, several attempts were required before the successful subculturing of strain F3-133^T^. The purity of the strain was subsequently checked by the colony morphology in the plate, microscopy and 16S rRNA gene amplification. Once its purity was confirmed, its genome was sequenced for further analyses.

### Genome-based phylogeny and taxonomy.

Complete 16S rRNA gene sequence (1470 nt) comparison of strain F3-133^T^ against available sequences in public databases revealed relative low relatedness (≤86.3% identity) with any previously cultivated microorganism. Strain F3-133^T^ proved to be most closely related to *Halobacteria* class representatives. Based on recently recommended values (e.g., 83 to 86% and 86 to 89% for the class-level and order-level classification, respectively [49, 50]), such 16S rRNA gene identity values indicate that strain F3-133^T^ represents a new order or even a new class within the phylum *Euryarchaeota*.

**FIG 1:**
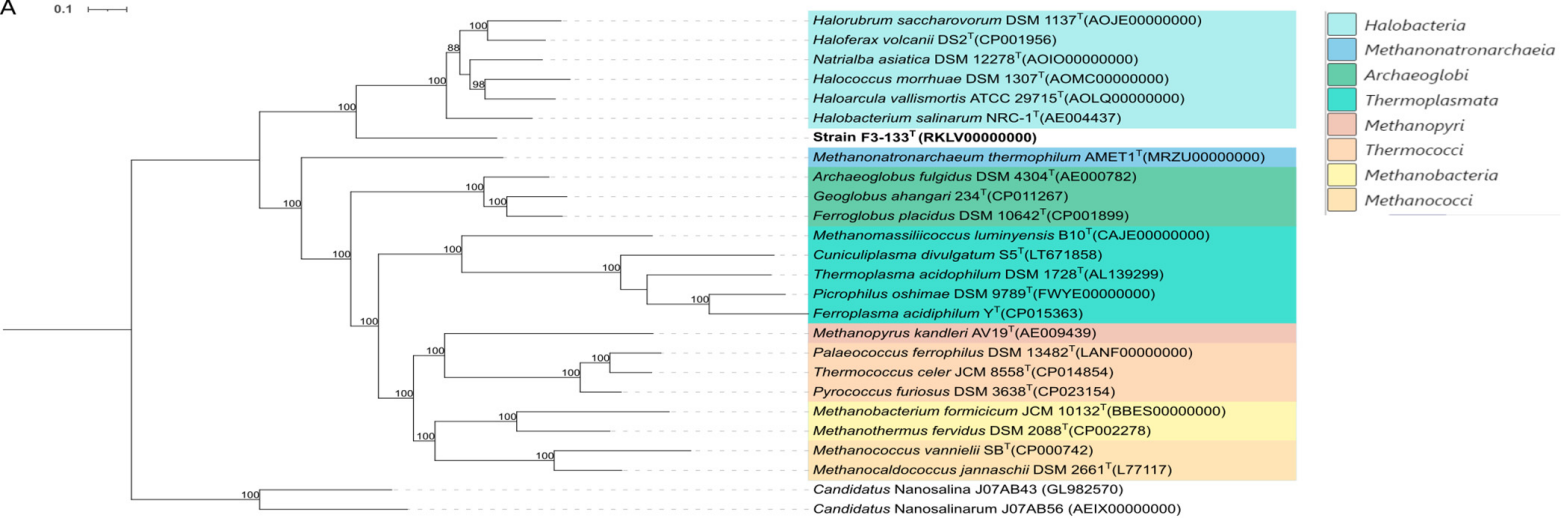

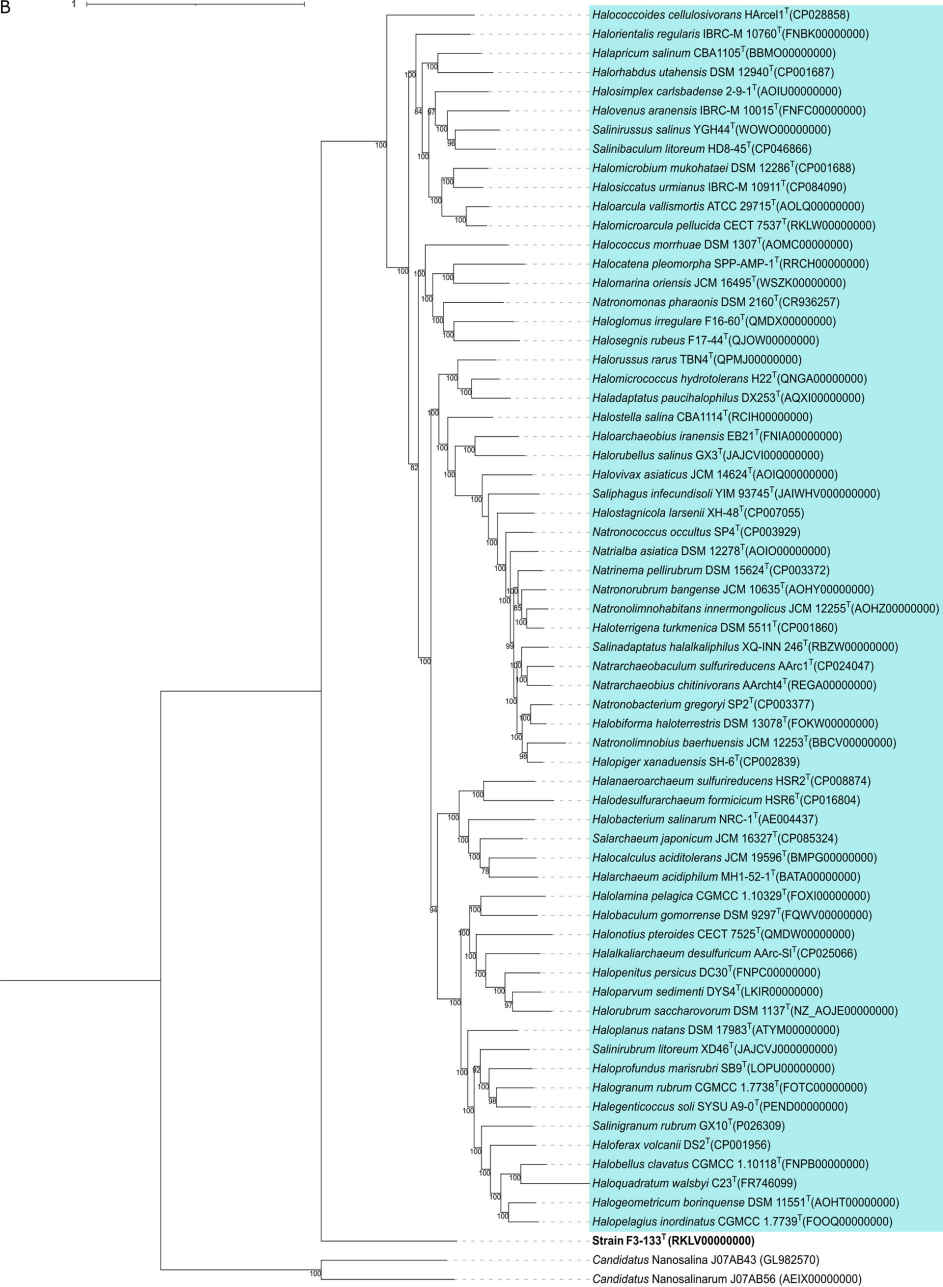
Approximate maximum-likelihood phylogenetic tree based on the concatenated amino acid sequence alignment of 62 (A) and 94 (B) single-copy genes. Shown are strain F3-133^T^ and the type strains of the type species of each type genus within all families of the class *Halobacteria* and representative members from the other classes of the phylum *Euryarchaeota* (A), and strain F3-133^T^ and the type strains of all type species within the class *Halobacteria* (B). The DPANN representatives *Candidatus* Nanosalinarum J07AB56 and *Candidatus* Nanosalina J07AB43 were used as outgroups. Genome accession numbers are shown in parentheses. Bootstrap values ≥70% are shown at branch points. Scale bars denotes 0.1 (A) and 1.0 (B) changes per nucleotide position.

**FIG 2:**
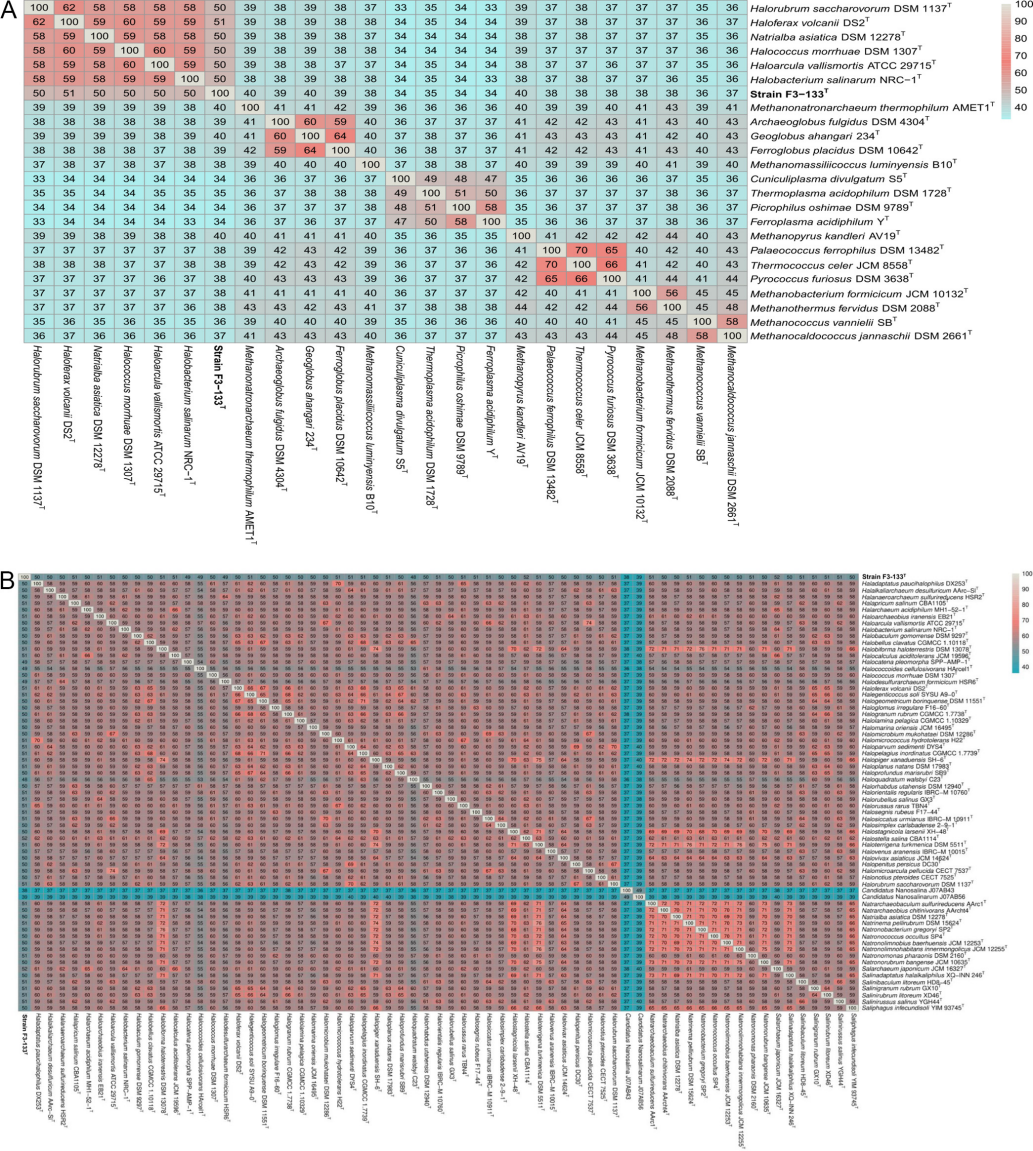
(A) Heatmap of AAI relatedness among strain F3-133^T^ and the type strains of all type genera of the class *Halobacteria* and representative members of the other classes of the phylum *Euryarchaeota*. (B) Heatmap of AAI relatedness among strain F3-133^T^ and the type strains of all type species within the class *Halobacteria* with available genomes. Genome accession numbers are specified on Fig. 1B and Table S4.

In order to further clarify the taxonomic position of this strain, a concatenated alignment of 62 single-copy genes shared between strain F3-133^T^ and representative members of different euryarchaeotal classes was used for an approximate maximum-likelihood tree reconstruction (Fig. 1A). Based on these results, strain F3-133^T^ represents an independent clade, a sister taxon to the class *Halobacteria*, with 100% bootstrap support (Fig. 1A). Consistent with this phylogeny-based picture, strain F3-133^T^ exhibited the highest AAI values (50 to 51%) to representatives of the class *Halobacteria* (Fig. 2A), with AAI values among *Halobacteria* members being substantially higher (58 to 62%) (Fig. 2A). Further comparison of strain F3-133^T^ with all type species from all orders and families of the class *Halobacteria* with available genomes supported its placement as a distinct phylogenomic branch within the class (Fig. 1B), which was also consistent with the AAI values among the same genomes (Fig. 2B). Collectively, it is clear enough that the evidence from 16S rRNA gene, universal protein-coding gene phylogenies and AAI analysis consistently and clearly support the proposal of strain F3-133^T^ as a novel order within the class *Halobacteria*, currently represented by a single family, genus, and species.

10.1128/msystems.01198-22.8 TABLE S4 Genomic size and DNA %G + C content of the type strains of the type species within the class *Halobacteria* with available genomes used for comparison in this study. Download Table S4, PDF file, 0.1 MB. Copyright © 2023 Durán-Viseras et al. 2023 Durán-Viseras et al. https://creativecommons.org/licenses/by/4.0/ This content is distributed under the terms of the Creative Commons Attribution 4.0 International license.

Furthermore, 16S rRNA gene phylogenetic analysis of strain F3-133^T^ and closely related environmental sequences from publicly available clone library data sets revealed that there might be other new species or genera within the proposed new family and order to be described in the future (Fig. S1). The genomic sequences of representatives of these groups will provide better resolution of the class status and a more thorough taxonomic description of its extant diversity in the future.

10.1128/msystems.01198-22.1 FIG S1 Maximum-likelihood phylogenetic tree reconstruction based on the 16S rRNA gene sequences of strain F3-133^T^, its related environmental high-quality sequences from SILVA database, and the type strain sequences from all type species of the class *Halobacteria*. For the environmental sequences, their sample origin (given in the sequence entry) and the sequence accession number are shown. Bootstrap values are indicated at branch points. Scale bar denotes 0.05 substitutions per nucleotide position. *Methanonatronarchaeum thermophilum* AMET1^T^ was used as outgroup. Download FIG S1, TIF file, 14.2 MB. Copyright © 2023 Durán-Viseras et al. 2023 Durán-Viseras et al. https://creativecommons.org/licenses/by/4.0/ This content is distributed under the terms of the Creative Commons Attribution 4.0 International license.

### Ecological distribution.

**FIG 3:**
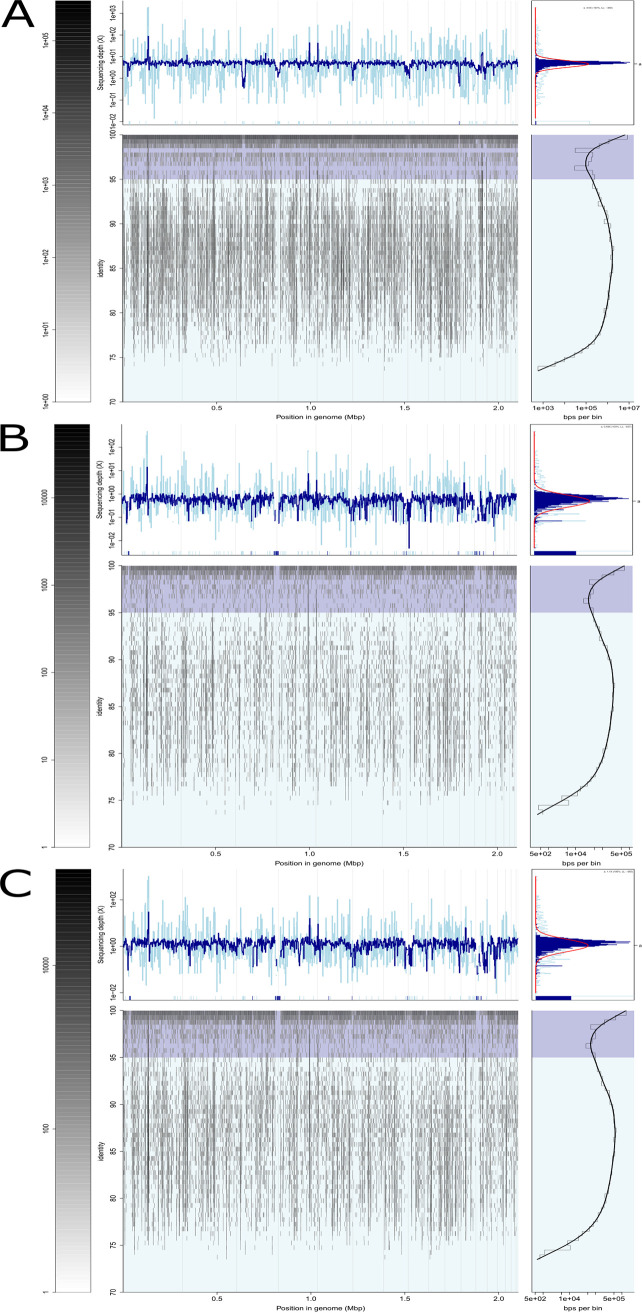
Read recruitment plot of metagenomic reads from (A) Isla Cristina solar saltern (PRJNA890281), (B) Velddrif solar saltern (ERR5981341), and (C) Chilean Altiplano lake against the genomic sequence of strain F3-133^T^. Within each plot: bottom middle panel represents the reads recruited, placed by position in the genome (X-axis) and percent of identity (Y-axis). Upper middle panel shows the sequencing depth per genome region in logarithmic scale. Bars at the bottom represent regions without mapping reads (sequencing depth of zero). Bottom right panel represents the number of nucleotide bases in logarithmic scale at each unit of nucleotide identity (*y* axis) in the bottom middle panel. Upper right panel exhibits the sequencing depth histogram across the genome. Left panel shows the color scale for the number of stacked reads per 2-dimensional bin in the bottom middle panel. The dark blue or light blue colors in the background of panels or the color lines correspond to matches with identity above and below 95% nucleotide identity cutoff.

To gain insight into the ecological distribution and relative abundance of strain F3-133^T^ in hypersaline environments from around the world, a large number of available metagenomic and 16S rRNA gene amplicons data sets were screened (Tables S1 and S2). Among ~ 200 metagenomes assessed (Table S2), only three provided significant read recruitment plots against the F3-133^T^ genome above the limit of detection of the metagenomic sequencing effort applied (Fig. 3). Specifically, F3-133^T^-like populations were present in the environment of origin, the brine of a pond of Isla Cristina saltern (42% total salts), as well as in a solar saltern in Velddrif (South Africa; 15% total salts) and a natural saline lake in the Chilean Altiplano (34% total salts) (Fig. 3), with a relative abundance of 0.1%, 0.03% and 0.02% of the total community, respectively. These results may indicate that strain F3-133^T^ increases its population abundance from non-detectable levels (rare biosphere) to an abundant member of the community under its optimal environmental conditions. Therefore, these results not only revealed that strain F3-133^T^ constitutes an abundant member of the microbial community in hypersaline environments widely distributed across the globe, but also reflected its versatility, being able to inhabit intermediate-to-high salinity systems. Moreover, the high number of reads recruiting below 95% identity in all three data sets suggested that there are likely additional, closely related, yet-to-be described species that are abundant (in order to be detected in the corresponding metagenomes) in these environments.

10.1128/msystems.01198-22.5 TABLE S1 16S rRNA gene amplicon data sets and clone library sequences analyzed in this study that contained related sequences to strain F3-133^T^. Download Table S1, PDF file, 0.1 MB. Copyright © 2023 Durán-Viseras et al. 2023 Durán-Viseras et al. https://creativecommons.org/licenses/by/4.0/ This content is distributed under the terms of the Creative Commons Attribution 4.0 International license.

10.1128/msystems.01198-22.6 TABLE S2 Metagenomic data sets used in this study for the recruitment plot analysis of strain F3-133^T^. Download Table S2, PDF file, 0.3 MB. Copyright © 2023 Durán-Viseras et al. 2023 Durán-Viseras et al. https://creativecommons.org/licenses/by/4.0/ This content is distributed under the terms of the Creative Commons Attribution 4.0 International license.

**FIG 4:**
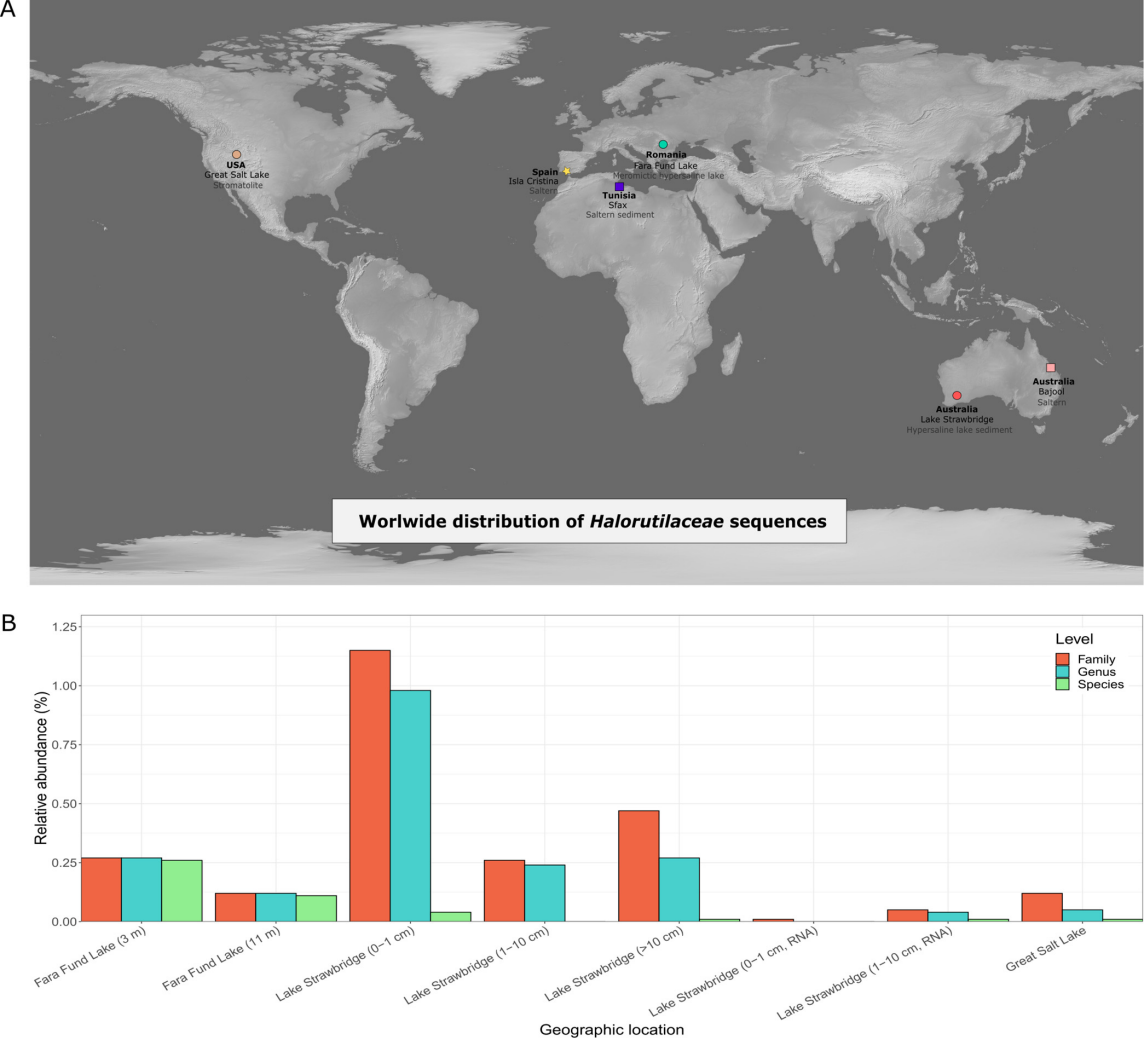
(A) Global distribution of strain F3-133^T^ and related organisms in hypersaline environments based on 16S rRNA gene amplicon and clone library data sets. Circles denote the geographic location of the related 16S rRNA gene sequences found in different amplicon data sets, while squares represent the sequences from clone library data sets. The star symbol indicates the place where strain F3-133^T^ was isolated. (B) Relative abundance of 16S rRNA gene sequences related to F3-133^T^ relative to all amplicon sequences in the corresponding data sets (at the geographic location highlighted with circles in [A]) assigned to the (same) family, genus, and species as strain F3-333^T^ based on identity threshold as described in the Materials and Methods section. Note that in some samples same-species sequences dominated the relatives found (e.g., Fara Fund Lake, Romania) while in some other samples the related sequences were mostly assigned to different species and genera than F3-133^T^ (e.g., Strawbridge Lake, Australia). RNA indicates transcriptomic data, while by default genomic data is used.

Further, screening of 16S rRNA gene amplicon or clone library data sets for close matches to the 16S rRNA gene sequence of F3-133^T^ revealed a remarkable number of sequences (1024 amplicons and 3 clones) related at the species (>98.6% identity), genus (>95% identity), and family (>92% identity) levels in samples from inland and coastal hypersaline environments in Australia, Romania, Tunisia, or USA (Fig. 4A). The salinities of those environments ranged from 5.1% total salts (Lake Strawbridge sediment) to 34% total salts (Bajool saltern) (Table S1), which are indeed comparable to the salinities of the metagenomes in which strain F3-133^T^ was found to be abundant (as specified above), further supporting the widespread presence of strain F3-133^T^ and related species in a broad range of salinity niches. With respect to 16S rRNA gene amplicon data set matches at genus level, the highest relative abundances were found in the sediments of Strawbridge Lake (Australia) (up to 0.9% of the total community) (Fig. 4B). However, the number of matches at species level in this environment were much fewer (Fig. 4B), reaching the highest relative abundance (0.3% of the total) in the meromictic hypersaline lake of Fara Fund (Romania). This fact, together with the metagenomic-based results mentioned above, enforce the hypothesis that aquatic niches are the preferred habitat for this group of microorganisms. Overall, these results further corroborated the worldwide distribution of F3-133^T^ and related species in a wide range of ecological niches associated with natural and manmade salterns, and suggested that more isolation efforts are needed to explore the extant diversity of this group of archaea.

### General genome characteristics.

The main genome characteristics of strain F3-133^T^ are provided in Table S3. One of the most remarkable features is the relatively small genome size of this new taxon (2.1 Mb) compared to other characterized halophilic archaeal species. To the best our knowledge, this is within the smallest genome sizes reported for any described member of the class *Halobacteria* to date (together with Halodesulfurarchaeum formicicum HSR6^T^, Halanaeroarchaeum sulfurireducens HSR2^T^, and Halobacterium salinarum NRC-1^T^ chromosome), with the available *Halobacteria* genomes to average ~ 4 Mb (Table S4). Small genomes in *Archaea* and *Bacteria* representatives are frequently associated with parasitic or symbiotic lifestyles, although free-living members in environments with low nutrient availability, such as aquatic ecosystems, can also exhibit small genomic sizes in comparison to nutrient-rich terrestrial ecosystems (51, 52). Moreover, smaller genome size species are often associated with an auxotrophic lifestyle (51, 53). These characteristics may also explain the preference of strain F3-133^T^ and relatives to aquatic environments (as discussed above), as well as its narrow nutritional requirements (strain F3-133^T^ can be only cultured in a defined medium with pyruvate, and is unable to use other compounds as carbon and energy sources) that presumably led to its fastidious growth during our culturomics isolation efforts. Further, autotrophy genes were also present in the F3-133^T^ genome, indicating that it can be truly free-living.

10.1128/msystems.01198-22.7 TABLE S3 Summary statistics of strain F3-133^T^ genome. Download Table S3, PDF file, 0.08 MB. Copyright © 2023 Durán-Viseras et al. 2023 Durán-Viseras et al. https://creativecommons.org/licenses/by/4.0/ This content is distributed under the terms of the Creative Commons Attribution 4.0 International license.

Moreover, a single rRNA operon was identified in the strain F3-133^T^ genome, consistent with an oligotrophic lifestyle (54). The presence of a single rRNA operon may also be related to the slow growth phylotype (54) exhibited by strain F3-133^T^, which also makes its cultivation under laboratory conditions challenging. Notably, the genome of strain F3-133^T^ is relatively low in DNA G+C content (59.8 mol%) in comparison with other halophilic archaea, which usually have G+C contents well above 60 mol% (Table S4), with the only known exceptions being Haloquadratum walsbyi (47.8 mol%), *Halocatena pleomorpha* SPP-AMP-1^T^ (57.1 mol%), Halonotius pteroides CECT 7525^T^ (59.5 mol%), and representatives of the *Candidatus* phylum Nanohaloarchaea (~ 40 mol%) (55).

The genome of strain F3-133^T^ revealed a very low percentage of non-coding sequences and an outstanding short median and average intergenic spacers (Fig. S2) in comparison to any previously cultivated *Halobacteria*. Altogether, these unique features indicate a streamlined genomic strategy for this strain (51, 56). Specifically, under some circumstances, selection may lead to a genomic complexity reduction in organisms that have large effective population sizes and resting cell stages or periods without significance growth, resulting in ecological successful strategies in nature, as for *Pelagibacter ubique* (57–59). Advances in multi-omics techniques revealed that the number of streamlined genomes in nature has been previously underestimated due to their fastidious growth under laboratory conditions (60). Besides, genome reduction is frequently associated with unusual nutritional requirements that necessitate specific cultivation approaches (56). In fact, strain F3-133^T^ was isolated using a culture medium similar to the ones used for growing other streamlined organisms. Indeed, the streamlined genome of strain F3-133^T^ probably accounts, at least in part, for the remarkable abundance and the worldwide-distribution of this new taxon in different hypersaline environments, but also to the previously unsuccessful attempts to isolate it. Lastly, small genome organisms are frequently related with relatively unchanging (stable) ecological niches, in contrast with fluctuating niches for which sigma factors play a crucial role (56). No sigma factors were identified in the isolate genome, corroborating its likely preferred distribution in aquatic ecosystems.

10.1128/msystems.01198-22.2 FIG S2 Scatter plots exhibiting the relation between length of intergenic regions and percentage of non-overlapping genes among strain F3-133^T^, and the type strain of all type species within the class *Halobacteria* with available genomes. Plots represent percentage of non-overlapping genes with respect to the average (A) and median (B) size of intergenic spacers, and to the average (C) and median (D) size of non-overlapping intergenic spacers. Genome accession numbers of *Halobacteria* representatives can be found on Table S4. Download FIG S2, TIF file, 10.2 MB. Copyright © 2023 Durán-Viseras et al. 2023 Durán-Viseras et al. https://creativecommons.org/licenses/by/4.0/ This content is distributed under the terms of the Creative Commons Attribution 4.0 International license.

### Central metabolism.

**FIG 5:**
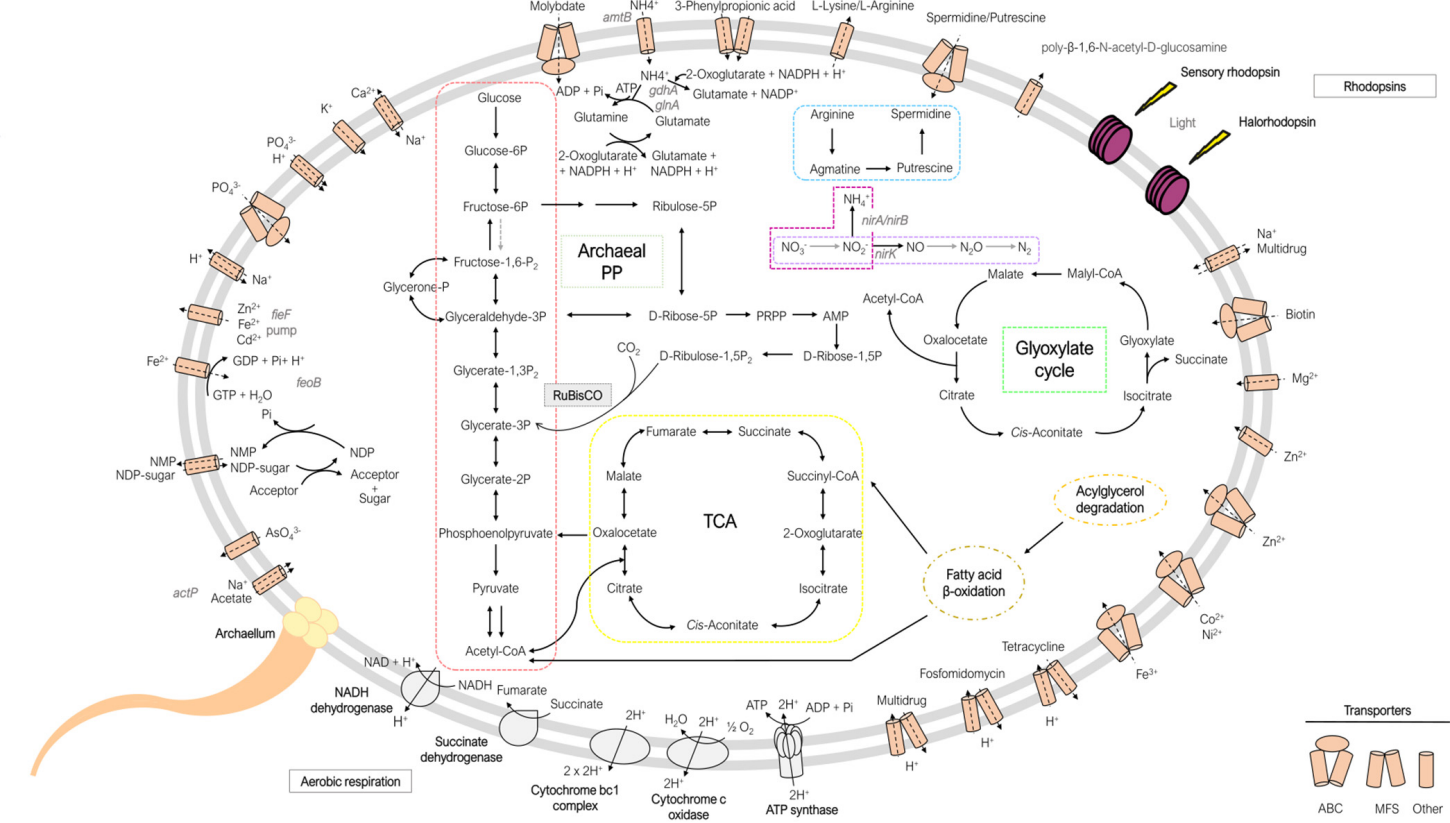
Genome scale metabolic reconstruction of strain F3-133^T^. The figure summarizes glycolysis/gluconeogenesis, pentose phosphate pathway, tricarboxylic acid cycle (TCA) autotrophic carbon fixation (AMP pathway), nitrogen metabolism (assimilatory and dissimilatory nitrate reduction, denitrification, and ammonia assimilation), polyamine biosynthesis, and glyoxylate cycle. Double lines surrounding the cell depict cell membrane. All predicted transporters with known functions are shown along the cell membrane and each family of transporter is displayed on the key. The arrows on the transporters indicate the flow direction (import, export, or symport). Within the glycolysis pathway, the gray discontinuous arrow indicates the gene absent.

Based on the bioinformatically annotated genome, strain F3-133^T^ encodes genes involved in gluconeogenesis, tricarboxylic acid cycle, and glyoxylate cycle (central carbohydrate metabolism; Fig. 5). However, as previously reported for other haloarchaea (40, 42, 61, 62), the gene coding for 6-phosphofructokinase involved in the lower part of the classical Embden–Meyerhof–Parnas (EM) pathway of glycolysis is absent in strain F3-133^T^, which could be alternatively replaced by the oxidative pentose phosphate pathway (Fig. 5) (63). Strain F3-133^T^ also possesses the pyruvate ferredoxin oxidoreductase genes (*porA* and *porB*) involved in the oxidation of pyruvate to acetyl-CoA (Fig. 5).

With respect to nitrogen metabolism, the genes encoding for nitrite reductases, i.e., nirA, nirB, and nirK, involved in assimilatory and dissimilatory nitrate reduction and denitrification, respectively, were all identified in the genome (Fig. 5). However, the remaining genes involved in these pathways, such as *narB*, *narGHI*, *norBC*, or *nosZ*, were not found, indicating that F3-133^T^ is probably doing only specific steps (modular) of denitrification. Strain F3-133^T^ also exhibited the high-affinity ammonium transporter (Amt) for ammonia uptake and the ABC transporter for the nitrogen derived compound spermidine (Fig. 5). The responsible genes for ammonia assimilation, glutamine synthetase, and glutamate synthase were also found in the genome (Fig. 5). Complete biosynthesis pathways of several amino acids (i.e., arginine, histidine, isoleucine, lysine, ornithine, proline, serine, threonine, and valine) were likewise identified.

Consistent with its physiological characterization, genes encoding archaellum were encountered in the genome (Fig. 5). Inorganic phosphate might be incorporated to the cell via an ABC transporter (pstSCAB). Rhodopsin-like sequences such as a sensory rhodopsin and a halorhodopsin were also identified in the genome (Fig. 5), suggesting a versatile metabolic flexibility under illuminated conditions. Additionally, strain F3-133^T^ possesses genes encoding enzymes of the fatty acid β-oxidation pathway (Fig. 5).

Strain F3-133^T^ encodes type III-like RuBisCO, and the associated AMP phosphorylase and ribose 1,5-biphosphate isomerase as part of the nucleotide monophosphate degradation pathway, which links nucleoside catabolism to glycolysis-gluconeogenesis (Fig. 5). The nucleotide monophosphate degradation pathway has been proposed in *Archaea* as part of a cyclic CO_2_ fixation pathway (61, 64), whereas this matter still remains unclear, and further examination will be necessary to understand its functional role in strain F3-133^T^. On the other side, this pathway has been suggested to be a relic of ancient heterotrophy considering ribose was likely the most abundant sugar available on early earth (65). The nucleotide monophosphate degradation pathway has been previously described for other archaeal representatives (i.e., representatives of the *Ca.* Asgardarchaeota, the methanogenic *Methanonatronarchaeia*, the hyperthermophilic archaeon Thermococcus kodakarensis, and the DPANN superphylum [66–69]). Although several halophilic archaea encode ribose 1,5-biphosphate isomerase (64), the evidence of type III-like RuBisCO presence has been only recently reported in members of this group (70); making strain F3-133^T^ one of the first known examples. Unexpectedly, strain F3-133^T^’s type III RuBisCO exhibited higher similarities to sequences from the archaeal phyla *Candidatus* Nanohaloarchaea or Division MSBL-1, than to *Halobacteria* class representatives. This fact reinforces the differences existing between strain F3-133^T^ and the *Halobacteria* class clade that, collectively with the already discussed distant phylogenomic position among them, supports the hypothesis that this new taxon might even constitute a different new class within the *Euryarchaeota* phylum. Future analyses and discovery of new representatives from this group would corroborate these results and interpretations.

### Adaptation to extreme salinity.

**FIG 6:**
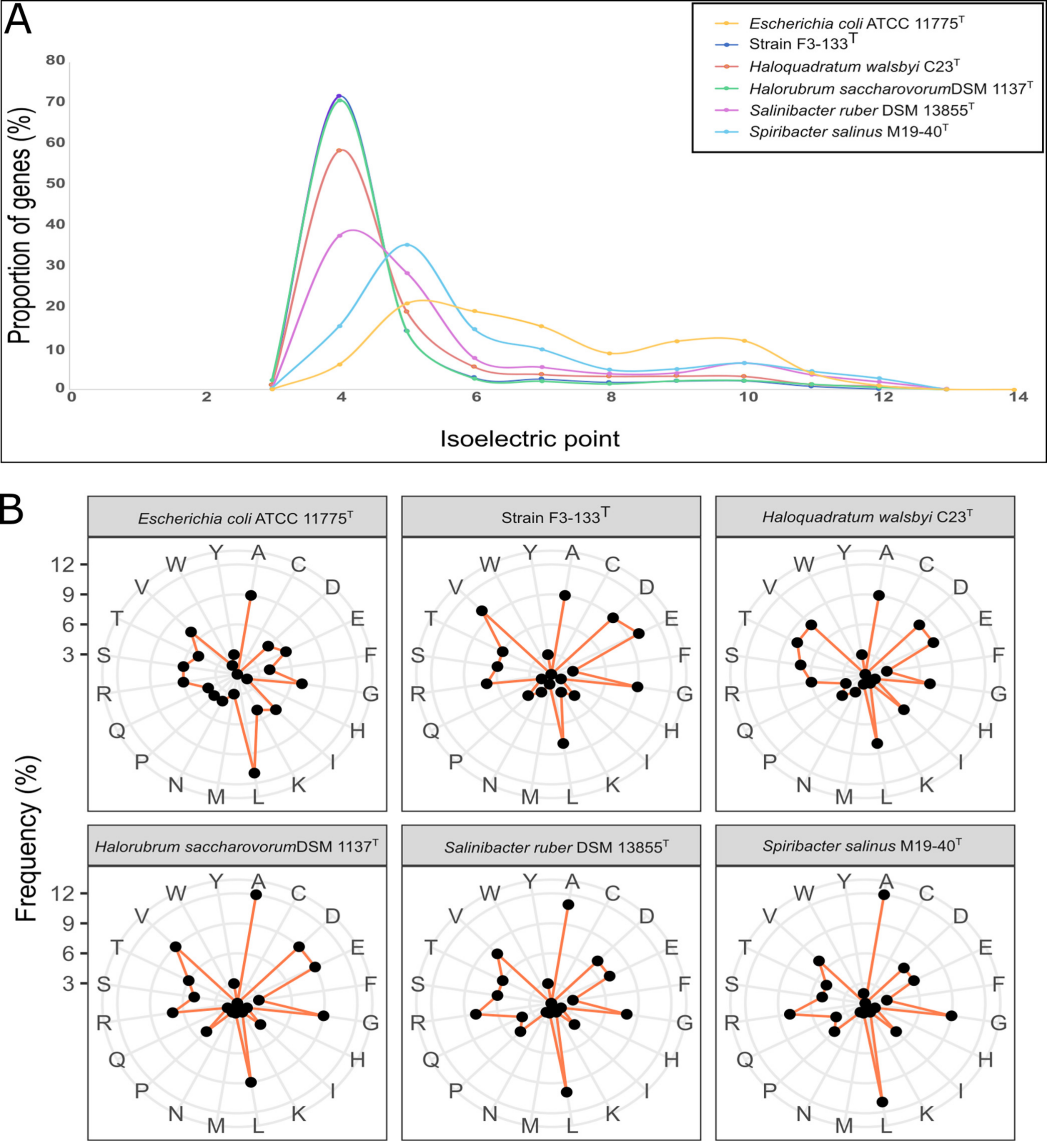
(A) Comparison of isoelectric point of predicted proteins for strain F3-133^T^ and selected genomes (graph title), shown as fraction of total genes (*y* axis). (B) Frequency of amino acids used in the proteome of strain F3-133^T^ and other selected genomes.

Considering the environmental distribution of strain F3-133^T^ and closely related sequences at different salinity ranges, the isoelectric points (pIs) and amino acid frequencies of the predicted proteins annotated in the genome of strain F3-133^T^ were calculated to shed light on its osmoregulatory adaptation mechanism. The proteome of the salt-in strategists Haloquadratum walsbyi, Halorubrum saccharovorum, or Salinibacter ruber, and the salt-out strategist Spiribacter salinus, and the non-halophile Escherichia coli were used for comparison. Results exhibited that the pI profile of strain F3-133^T^ was similar to the *salt-in Halobacteria* representatives and Salinibacter ruber, with a single peak at around 4.0, pointing out the predominance of acidic residues (Fig. 6A). Accordingly, a prevalence of acidic amino acids (i.e., aspartate and glutamate) in comparison to basic amino acids (i.e., arginine and lysine) was observed for strain F3-133^T^, as well as for the other *salt-in* strategists analyzed (Fig. 6B). Therefore, it appears that strain F3-133^T^ employs the *salt-in* strategy for balancing the osmotic pressure of the saline environments.

In good agreement with a (hypothesized) *salt-in* osmoprotection mechanism (71, 72), several genes encoding for secondary transporters involved in Na^+^ extrusion, K^+^ uptake, and Cl^−^ homeostasis were also found in the genome of strain F3-133^T^ (Fig. 5). Moreover, no genes related to the biosynthesis or transport of compatible solutes were identified, consistent with the hypothesis that a *salt-out* strategy is not used by strain F3-133^T^. Lastly, a small conductance mechanosensitive channel from the MscS family, which confers significant protection against rapid hypoosmotic shock (i.e., rapid transition from high to moderate salinity environments) (73), was also detected in the genome.

### Physiology of strain F3-133^T^.

We carried out a detailed phenotypic characterization of the new isolate following the recommended minimal standards for describing new taxa of the class *Halobacteria* (74). These features included morphological, physiological, biochemical, and nutritional characteristics, as well as the determination of the membrane polar lipids, which has been proved to be an important feature for the characterization of haloarchaeal genera (74, 75). These results are shown in Table S5, Fig. S3 and S4, and in the new genus and species descriptions detailed below. Overall, all these results are in good agreement with the metabolic behavior identified by the genomic and metagenomic analysis, such as preference and tolerance for a range of salt concentrations, and the stringent nutritional requirements. Notably, strain F3-133^T^ exhibits a very simplified chemotaxonomic profile, which is also consistent with its distant phylogenomic relationship with other members of the class *Halobacteria*. Further, the presence of the double chain length C_20_,C_25_ derived from PGP-Me as chemotaxonomic feature (Fig. S4) is not typically found in neutrophilic haloarchaeal species, which might also suggest the adaptability of this strain to thrive in environments with pH fluctuation. In the same way, the absence of glycolipids, as in strain F3-133^T^ (Fig. S4), is a typical chemotaxonomic characteristic of haloalkaliphilic archaea. Indeed, glycolipids are associated with bacteriorhodopsin sequences, which were consistently absent in the genome of strain F3-133^T^ (Fig. 5).

10.1128/msystems.01198-22.3 FIG S3 Phase-contrast photomicrograph of cells of strain F3-133^T^ cultured in liquid medium under optimal conditions. Scale bar denotes 10 μm. Download FIG S3, TIF file, 13.2 MB. Copyright © 2023 Durán-Viseras et al. 2023 Durán-Viseras et al. https://creativecommons.org/licenses/by/4.0/ This content is distributed under the terms of the Creative Commons Attribution 4.0 International license.

10.1128/msystems.01198-22.4 FIG S4 High performance thin layer chromatography (HPTLC) exhibiting the polar lipids profile of strain F3-133^T^ in comparison to reference haloarchaeal species. The plate was revealed with 5% H_2_SO_4_ (in water) as spray reagent followed by heating at 160°C. BPG, biphosphatidylglycerol; PG, phosphatidylglycerol; PGP-Me, phosphatidylglycerol phosphate methyl ester; PGS, phosphatidylglycerol sulfate; S-DGD-1, sulphated diglycosyl diether; S-TGD-1-PA, glycocardiolipin (sulfated triglycosyl diphytanyl archaeol ester linked to phosphatidic acid); S-TeGD, sulphated tetraglycosyl diether. Download FIG S4, TIF file, 13.1 MB. Copyright © 2023 Durán-Viseras et al. 2023 Durán-Viseras et al. https://creativecommons.org/licenses/by/4.0/ This content is distributed under the terms of the Creative Commons Attribution 4.0 International license.

10.1128/msystems.01198-22.9 TABLE S5 Phenotypic features of strain F3-133^T^. Download Table S5, PDF file, 0.2 MB. Copyright © 2023 Durán-Viseras et al. 2023 Durán-Viseras et al. https://creativecommons.org/licenses/by/4.0/ This content is distributed under the terms of the Creative Commons Attribution 4.0 International license.

Based on the taxogenomic comparison of strain F3-133^T^ to available genomes and its physiology, we propose a new order, family, genus, and species within the class *Halobacteria* with the names *Halorutilales* ord. nov., *Halorutilaceae* fam. nov., *Halorutilus* gen. nov., and *Halorutilus salinus* sp. nov. to accommodate the new isolate as detailed below.

### Description of *Halorutilus* gen. nov.

*Halorutilus* (Ha.lo.ru.ti’lus. Gr masc. n. *hals*, *halos* salt; L. masc. adj. *rutilus* red; N.L. masc. n. *Halorutilus* a red-pigmented halophilic/salt microorganism).

Cells are motile, short rods (0.4-0.7 × 1.1-1.4 μm) producing intense red-pigmented colonies. Extremely halophilic and aerobic. Photoheterotrophic metabolism. Catalase positive but oxidase negative. The polar lipids are phosphatidylglycerol (PG), phosphatidylglycerol phosphate methyl ester (PGP-Me), and one phospholipid chromatographically identical to sulfated mannosyl glucosyl diether (S-DGD-1).

The type species is *Halorutilus salinus*. The DNA G+C content is 59.8 mol% (genome). Phylogenetically affiliated to the class *Halobacteria*. The genome size is 2.1 Mb. Recommended 3 letter abbreviation: Hrt.

### Description of *Halorutilus salinus* sp. nov.

*Halorutilus salinus* (sa .li'nusnus. N.L. masc. adj. *salinus*, salted, salty).

Cells are motile, short rods and pleomorphic (0.4-0.7 × 1.1-1.4 μm) (Fig. S3). Colonies are circular, entire, intense red-pigmented with a diameter of 1.5 mm on solid 25% salts medium after 21 days at 37°C. Growth occurs optimally at 25% (wt/vol) NaCl, pH 7.5, and 37°C. Nitrate and nitrite are reduced. The DNA G+C content is 59.8 mol% (genome). Other features are detailed in the genus description and in Table S5.

The type strain is F3-133^T^ (= CCM 9157^T^ = JCM 33312^T^), isolated from a water sample of a pond from Isla Cristina saltern (Huelva, Spain).

The GenBank/EMBL/DDBJ accession number for the 16S rRNA gene sequence of *Halorutilus salinus* F3-133^T^ is MK182271, and that of the complete genome is RKLV00000000.

### Description of *Halorutilaceae* fam. nov.

*Halorutilaceae* (Ha.lo.ru.ti.la.ce’ae. N.L. masc. n. *Halorutilus* the type genus of the family; L. suff. -aceae ending to denote a family; N.L. fem. pl. n. *Halorutilaceae* the family the nomenclatural type of which is the genus *Halorutilus*).

The properties of the family are the same as for the representative genus *Halorutilus*. Currently, the family is monotypic and the type genus is *Halorutilus*. Phylogenetically affiliated to the class *Halobacteria*.

### Description of *Halorutilales* ord. nov.

*Halorutilales* (Ha.lo.ru.ti.la’les. N.L. masc. n. *Halorutilus* the type genus of the order; L. fem. pl. n. suff. -ales ending to denote an order; N.L. fem. pl. n. *Halorutilales* the order the nomenclatural type of which is the genus *Halorutilus*).

The properties of the order *Halorutilales* are the same as for the representative genus *Halorutilus*. The type genus of the order is *Halorutilus*.

## MATERIALS AND METHODS

### Isolation source and culture conditions of strain F3-133^T^.

Strain F3-133^T^ was isolated from a pond water sample (23% wt/vol salinity and pH 7.5) collected from the Isla Cristina solar saltern (Huelva, Spain, 37°21’ N 7°33’W) during an extensive sampling campaign carried out in June 2016. Following the culturomics methodology previously described (40), and after several attempts, strain F3-133^T^ was obtained in pure culture on a solid medium containing the following salt mixture (g/L): NaCl, 195; MgCl_2_·6H_2_O, 32.5; MgSO_4_·7H_2_O, 50.8; CaCl_2_, 0.83; KCl, 5.0; NaHCO_3_, 0.21; NaBr, 0.58, and supplemented with pyruvate (1 g/L) and casein digest (5 g/L). The pH was adjusted to 7.5, and purified agar (Oxoid) was added when necessary. This medium was also used for routine growth of the strain. For long-term preservation cultures were maintained at −80°C in this medium containing 20% (vol/vol) glycerol.

### Physiological characterization.

Phenotypic features of strain F3-133^T^ were performed following the methodology previously described by Durán-Viseras et al. (43), and according to the minimal standards established for the taxonomic description of novel taxa of the class *Halobacteria* (74). Determination of the polar lipid profile of strain F3-133^T^ was carried out by high performance thin-layer chromatography (HPTLC) and revealed using as spray reagents 5% H_2_SO_4_ (in water) (43). Halobacterium salinarum DSM 3754^T^ and Halorubrum saccharovorum DSM 1137^T^ were used as reference species for polar lipids characterization.

### Genomic DNA extraction, sequencing, and data curation.

Genomic DNA of strain F3-133^T^ was extracted, purified, and quantified following the methodology previously described by Durán-Viseras et al. (39). The 16S rRNA gene was amplified by PCR using the primer pairs ArchF/ArchR (76, 77) and sequenced by Sanger technology at StabVida (Caparica, Portugal). The whole genome shotgun sequencing of strain F3-133^T^ was also performed by StabVida (Caparica, Portugal) using a 150 bp paired-end sequencing strategy, on the Illumina HiSeq 4000 platform. Sequencing reads were quality filtered and trimmed using BBTools v.38.44 (https://sourceforge.net/projects/bbmap/), and then assembled with SPAdes v.3.12.0 (78). CheckM v1.0.5 (79) and Quast v2.3 (80) were used for assembly quality checks. Genes were predicted on the assembled contigs using Prodigal (81), and the predicted genes were then annotated with Prokka (82). BlastKOALA (83) was used to assign KO identifiers (K numbers) to orthologous genes present in the genomes analyzed and, subsequently, mapped to the KEGG pathways and KEGG modules (84) in order to perform metabolic pathway reconstructions. The Average Amino acid Identity (AAI) was determined for all-versus-all genome pairs using the tool AAI-Matrix from the Enveomics collection (85). Isoelectric points and amino acid frequencies of predicted proteins were estimated using the tools iep and pepstats, respectively, from the EMBOSS package (86).

### 16S rRNA gene and universal protein-coding gene phylogenetic analysis.

The 16S rRNA gene sequence of strain F3-133^T^ was compared to the sequences in the EzBioCloud server (87) and SILVA REF 138 database (88) to get the sequence identity to previously described taxa, and to identify related sequences for phylogenetic comparisons. The 16S rRNA gene sequences of the type strains of the type species of all genera of the class *Halobacteria*, and the identified related sequences were downloaded from GenBank/EMBL/DDBJ and SILVA databases, respectively.

For the 16S rRNA gene-based phylogenetic analysis, almost complete 16S rRNA gene sequences were aligned using the software MAFFT (89). Subsequently, the phylogenetic tree was constructed with the RaxML software (90).

For the phylogenomic analysis, core orthologous genes were determined using an all-versus-all BLASTp comparison among the translated CDS features of the annotated genomes under study, as implemented in the Enveomics collection toolbox (85). Afterwards, the translated single-copy core gene sequences were individually aligned with Muscle (91) and concatenated into a super-protein alignment. FastTreeMP v.2.1.8 (92) was used for the phylogenomic tree reconstruction by means of the approximately maximum-likelihood algorithm.

The online tool iTOL v.5.7 (93) was used to display the 16S rRNA gene-based and phylogenomic trees.

### Ecological prevalence and estimated abundance of strain F3-133^T^ in saline habitats.

The abundance of strain F3-133^T^ in available metagenomes was assessed by read fragment recruitment plots (94). For this, the genome sequence of strain F3-133^T^ was searched against all metagenomic reads from each data set (Table S2) using stand-alone BLASTn. Read recruitment plots were constructed using the enveomics collection (85) based on the best BLAST best-matches (with a cutoff 70% query coverage).

The identification and geographical distribution based on 16S rRNA gene data was performed as follows: firstly, the online tool IMNGS (95) was used to identify the publicly available amplicon project data sets containing 16S rRNA gene sequences related to strain F3-133^T^. The 16S rRNA gene of strain F3-133^T^ was screened against all amplicon project data sets (Table S1) using BLASTn. Only BLAST best-matches with a minimum query size of 250 bp, 70% query coverage and identity ≥92, 95 and 98.6% (for family, genus, and species level relatedness, respectively) were considered. The geographical location of the data sets that provided matching sequences are displayed on the map (Fig. 4A), and the relative abundances of matches at family, genus, and species level relatedness in each data set is shown in Fig. 4B.

## References

[1] Edgerton ME, Brimblecombe P. 1981. Thermodynamics of halobacterial environments. Can J Microbiol 27:899–909. doi:10.1139/m81-142. 7306878

[2] Rodríguez-Valera F. 1988. Characteristics and microbial ecology of hypersaline environments, p 3–30. *In* Rodríguez-Valera F (ed), Halophilic bacteria. CRC Press, Inc., Boca Raton, FL.

[3] Oren A. 2014. Halophilic archaea on Earth and in space: growth and survival under extreme conditions. Philos Trans R Soc A 372:20140194. doi:10.1098/rsta.2014.0194. 25368347

[4] Fox-Powell MG, Hallsworth JE, Cousins CR, Cockell CS. 2016. Ionic strength is a barrier to the habitability of Mars. Astrobiology 16:427–442. doi:10.1089/ast.2015.1432. 27213516

[5] Pontefract A, Zhu TF, Walker VK, Hepburn H, Lui C, Zuber MT, Ruvkun G, Carr CE. 2017. Microbial diversity in a hypersaline sulfate lake: a terrestrial analog of ancient mars. Front Microbiol 8:1819. doi:10.3389/fmicb.2017.01819. 29018418 PMC5623196

[6] Oren A. 2010. Industrial and environmental applications of halophilic microorganisms. Environ Technol 31:825–834. doi:10.1080/09593330903370026. 20662374

[7] Amoozegar MA, Siroosi M, Atashgahi S, Smidt H, Ventosa A. 2017. Systematics of haloarchaea and biotechnological potential of their hydrolytic enzymes. Microbiology 163:623–645. doi:10.1099/mic.0.000463. 28548036

[8] Corral P, Amoozegar MA, Ventosa A. 2020. Halophiles and their biomolecules: recent advances and future applications in biomedicine. Mar Drugs 18:33. doi:10.3390/md18010033. PMC7024382 31906001

[9] Mesbah NM, Wiegel J. 2012. Life under multiple extreme conditions: diversity and physiology of the halophilic alkalithermophiles. Appl Environ Microbiol 78:4074–4082. doi:10.1128/AEM.00050-12. 22492435 PMC3370554

[10] Gomariz M, Martínez-García M, Santos F, Rodriguez F, Capella-Gutiérrez S, Gabaldón T, Rosselló-Móra R, Meseguer I, Antón J. 2015. From community approaches to single-cell genomics: the discovery of ubiquitous hyperhalophilic *Bacteroidetes* generalists. ISME J 9:16–31. doi:10.1038/ismej.2014.95. 24926861 PMC4274432

[11] Mora-Ruiz M del R, Cifuentes A, Font-Verdera F, Pérez-Fernández C, Farias ME, González B, Orfila A, Rosselló-Móra R. 2018. Biogeographical patterns of bacterial and archaeal communities from distant hypersaline environments. Syst Appl Microbiol 41:139–150. doi:10.1016/j.syapm.2017.10.006. 29352612

[12] Viver T, Orellana L, González-Torres P, Díaz S, Urdiain M, Farías ME, Benes V, Kaempfer P, Shahinpei A, Amoozegar MA, Amann R, Antón J, Konstantinidis KT, Rosselló-Móra R. 2018. Genomic comparison between members of the *Salinibacteraceae* family, and description of a new species of *Salinibacter* (*Salinibacter altiplanensis* sp. nov.) isolated from high altitude hypersaline environments of the Argentinian Altiplano. Syst Appl Microbiol 41:198–212. doi:10.1016/j.syapm.2017.12.004. 29429564

[13] Viver T, Orellana LH, Díaz S, Urdiain M, Ramos‐Barbero MD, González‐Pastor JE, Oren A, Hatt JK, Amann R, Antón J, Konstantinidis KT, Rosselló‐Móra R. 2019. Predominance of deterministic microbial community dynamics in salterns exposed to different light intensities. Environ Microbiol 21:4300–4315. doi:10.1111/1462-2920.14790. 31444990

[14] Oren A. 2014. Taxonomy of halophilic Archaea: current status and future challenges. Extremophiles 18:825–834. doi:10.1007/s00792-014-0654-9. 25102811

[15] Oren A, Ventosa A, Kamekura M. 2017. Halobacteria. Bergey’s manual of systematics of Archaea and Bacteria. John Wiley & Sons, Inc., West Sussex, UK.

[16] Ventosa A. 2006. Unusual micro-organisms from unusual habitats: hypersaline environments, p 223–254. *In* Prokaryotic diversity: mechanisms and significance. Cambridge University Press, Cambridge UK.

[17] Oren A. 2011. Ecology of halophiles, p 343–361. *In* Horikoshi K (ed), Extremophiles handbook. Springer Japan, Tokyo, Japan.

[18] Parte AC, Sardà Carbasse J, Meier-Kolthoff JP, Reimer LC, Göker M. 2020. List of prokaryotic names with standing in nomenclature (LPSN) moves to the DSMZ. Int J Syst Evol Microbiol 70:5607–5612. doi:10.1099/ijsem.0.004332. 32701423 PMC7723251

[19] Oren A, Ventosa A. 2019. International committee on systematics of prokaryotes subcommittee on the taxonomy of *Halobacteria* and subcommittee on the taxonomy of *Halomonadaceae*. Minutes of the joint open meeting, 26 June 2019, Cluj-Napoca, Romania. Int J Syst Evol Microbiol 69:3657–3661. doi:10.1099/ijsem.0.003737. 31526449

[20] Gupta RS, Naushad S, Baker S. 2015. Phylogenomic analyses and molecular signatures for the class *Halobacteria* and its two major clades: a proposal for division of the class *Halobacteria* into an emended order *Halobacteriales* and two new orders, *Haloferacales* ord. nov. and *Natrialbales* ord. nov. Int J Syst Evol Microbiol 65:1050–1069. doi:10.1099/ijs.0.070136-0. 25428416

[21] Gupta RS, Naushad S, Fabros R, Adeolu M. 2016. A phylogenomic reappraisal of family-level divisions within the class *Halobacteria*: proposal to divide the order *Halobacteriales* into the families *Halobacteriaceae*, *Haloarculaceae* fam. nov., and *Halococcaceae* fam. nov., and the order *Haloferacales* into the families *Haloferacaceae* and *Halorubraceae* fam. nov. Antonie van Leeuwenhoek 109:565–587. doi:10.1007/s10482-016-0660-2. 26837779

[22] Wu S, Wang J, Wang J, Du X, Ran Q, Chen Q, Sheng D, Li Y-Z. 2022. *Halalkalibacterium roseum* gen. nov., sp. nov., a new member of the family *Balneolaceae* isolated from soil. Int J Syst Evol Microbiol 72:e005339. doi:10.1099/ijsem.0.005339. 35482520

[23] Zhu K-L, Wang X-Q, Zhang T-S, Shang D-D, Du Z-J. 2021. *Salibaculum halophilum* gen. nov., sp. nov. and *Salibaculum griseiflavum* sp. nov., in the family *Rhodobacteraceae*. Int J Syst Evol Microbiol 71:e004808. doi:10.1099/ijsem.0.004808. 34170216

[24] Zuo Z, Zhao D, Zhou J, Han J, Xiang H. 2021. *Halalkalirubrum salinum* gen. nov., sp. nov., a halophilic archaeon isolated from a saline lake. Antonie van Leeuwenhoek 114:83–94. doi:10.1007/s10482-020-01502-6. 33389352

[25] Parada-Pinilla MP, Díaz-Cárdenas C, López G, Díaz-Riaño JI, Gonzalez LN, Restrepo S, Trujillo ME, Baena S. 2020. *Salifodinibacter halophilus* gen. nov., sp. nov., a halophilic gammaproteobacterium in the family *Salinisphaeraceae* isolated from a salt mine in the Colombian Andes. Int J Syst Evol Microbiol 70:5888–5898. doi:10.1099/ijsem.0.004490. 33034549

[26] Durán-Viseras A, Sánchez-Porro C, Ventosa A. 2020. *Haloglomus irregulare* gen. nov., sp. nov., a new halophilic archaeon isolated from a marine saltern. Microorganisms 8:206. doi:10.3390/microorganisms8020206. 32024278 PMC7074781

[27] Chen S, Xu Y, Sun S, Liu J, Chen F. 2020. *Halomicrococcus hydrotolerans* gen. nov., sp. nov., an extremely halophilic archaeon isolated from a subterranean salt deposit. Int J Syst Evol Microbiol 70:4425–4431. doi:10.1099/ijsem.0.003534. 31204974

[28] Sorokin DY, Yakimov M, Messina E, Merkel AY, Bale NJ, Sinninghe Damsté JS. 2019. *Natronolimnobius sulfurireducens* sp. nov. and *Halalkaliarchaeum desulfuricum* gen. nov., sp. nov., the first sulfur-respiring alkaliphilic haloarchaea from hypersaline alkaline lakes. Int J Syst Evol Microbiol 69:2662–2673. doi:10.1099/ijsem.0.003506. 31166158

[29] Sorokin DY, Elcheninov AG, Toshchakov SV, Bale NJ, Sinninghe Damsté JS, Khijniak TV, Kublanov IV. 2019. *Natrarchaeobius chitinivorans* gen. nov., sp. nov., and *Natrarchaeobius halalkaliphilus* sp. nov., alkaliphilic, chitin-utilizing haloarchaea from hypersaline alkaline lakes. Syst Appl Microbiol 42:309–318. doi:10.1016/j.syapm.2019.01.001. 30638904 PMC6542413

[30] Sorokin DY, Khijniak TV, Elcheninov AG, Toshchakov SV, Kostrikina NA, Bale NJ, Sinninghe Damsté JS, Kublanov IV. 2019. *Halococcoides cellulosivorans* gen. nov., sp. nov., an extremely halophilic cellulose-utilizing haloarchaeon from hypersaline lakes. Int J Syst Evol Microbiol 69:1327–1335. doi:10.1099/ijsem.0.003312. 30801242

[31] Ghai R, Pašić L, Fernández AB, Martin-Cuadrado AB, Mizuno CM, McMahon KD, Papke RT, Stepanauskas R, Rodriguez-Brito B, Rohwer F, Sánchez-Porro C, Ventosa A, Rodríguez-Valera F. 2011. New abundant microbial groups in aquatic hypersaline environments. Sci Rep 1:135. doi:10.1038/srep00135. 22355652 PMC3216616

[32] Fernández AB, Ghai R, Martín-Cuadrado AB, Sánchez-Porro C, Rodríguez-Valera F, Ventosa A. 2014. Prokaryotic taxonomic and metabolic diversity of an intermediate salinity hypersaline habitat assessed by metagenomics. FEMS Microbiol Ecol 88:623–635. doi:10.1111/1574-6941.12329. 24661078

[33] Vera-Gargallo B, Ventosa A. 2018. Metagenomic insights into the phylogenetic and metabolic diversity of the prokaryotic community dwelling in hypersaline soils from the Odiel Saltmarshes (SW Spain). Genes 9:152. doi:10.3390/genes9030152. 29518047 PMC5867873

[34] Spang A, Caceres EF, Ettema TJG. 2017. Genomic exploration of the diversity, ecology, and evolution of the archaeal domain of life. Science 357:eaaf3883. doi:10.1126/science.aaf3883. 28798101

[35] Narasingarao P, Podell S, Ugalde JA, Brochier-Armanet C, Emerson JB, Brocks JJ, Heidelberg KB, Banfield JF, Allen EE. 2012. *De novo* metagenomic assembly reveals abundant novel major lineage of Archaea in hypersaline microbial communities. ISME J 6:81–93. doi:10.1038/ismej.2011.78. 21716304 PMC3246234

[36] Podell S, Ugalde JA, Narasingarao P, Banfield JF, Heidelberg KB, Allen EE. 2013. Assembly-driven community genomics of a hypersaline microbial ecosystem. PLoS One 8:e61692. doi:10.1371/journal.pone.0061692. 23637883 PMC3630111

[37] Vartoukian SR, Palmer RM, Wade WG. 2010. Strategies for culture of ‘unculturable’ bacteria. FEMS Microbiol Lett 309:1–7. doi:10.1111/j.1574-6968.2010.02000.x. 20487025

[38] Lagier J-C, Khelaifia S, Tidjani Alou M, Ndongo S, Dione N, Hugon P, Caputo A, Cadoret F, Ibrahima Traore S, Hadji Seck E, Dubourg G, Durand G, Mourembou G, Guilhot E, Togo A, Bellali S, Bachar D, Cassir N, Bittar F, Delerce J, Mailhe M, Ricaboni D, Bilen M, Prisca Makaya Dangui Nieko N, Mery Dia Badiane N, Valles C, Mouelhi D, Diop K, Million M, Musso D, Abrahão J, Ibraheem Azhar E, Bibi F, Yasir M, Diallo A, Sokhna C, Djossou F, Vitton V, Robert C, Marc Rolain J, La Scola B, Fournier P-E, Levasseur A, Raoult D. 2016. Culture of previously uncultured members of the human gut microbiota by culturomics. Nat Microbiol 1:16203. doi:10.1038/nmicrobiol.2016.203. 27819657 PMC12094094

[39] Durán-Viseras A, Sánchez-Porro C, Ventosa A. 2021. Genomic insights into new species of the genus *Halomicroarcula* reveals potential for new osmoadaptative strategies in halophilic archaea. Front Microbiol 12:1–16. doi:10.3389/fmicb.2021.751746. PMC8600319 34803972

[40] Durán-Viseras A, Andrei AŞ, Vera-Gargallo B, Ghai R, Sánchez-Porro C, Ventosa A. 2021. Culturomics-based genomics sheds light on the ecology of the new haloarchaeal genus *Halosegnis*. Environ Microbiol 23:3418–3434. doi:10.1111/1462-2920.15082. 32410366

[41] Durán-Viseras A, Ventosa A, Sánchez-Porro C. 2019. *Halonotius aquaticus* sp. nov., a new haloarchaeon isolated from a marine saltern. Int J Syst Evol Microbiol 69:1306–1312. doi:10.1099/ijsem.0.003309. 30789324

[42] Durán-Viseras A, Andrei A-S, Ghai R, Sánchez-Porro C, Ventosa A. 2019. New *Halonotius* species provide genomics-based insights into cobalamin synthesis in haloarchaea. Front Microbiol 10:1928. doi:10.3389/fmicb.2019.01928. 31507553 PMC6719526

[43] Durán-Viseras A, Sánchez-Porro C, Ventosa A. 2020. *Natronomonas salsuginis* sp. nov., a new inhabitant of a marine solar saltern. Microorganisms 8:605. doi:10.3390/microorganisms8040605. 32326357 PMC7232251

[44] Akpolat C, Fernández AB, Caglayan P, Calli B, Birbir M, Ventosa A. 2021. Prokaryotic communities in the thalassohaline Tuz Lake, deep zone, and Kayacik, Kaldirim and Yavsan Salterns (Turkey) assessed by 16S rRNA amplicon sequencing. Microorganisms 9:1525. doi:10.3390/microorganisms9071525. 34361960 PMC8304926

[45] Jookar Kashi F, Owlia P, Amoozegar MA, Kazemi B. 2021. Halophilic prokaryotes in Urmia Salt Lake, a hypersaline environment in Iran. Curr Microbiol 78:3230–3238. doi:10.1007/s00284-021-02583-w. 34216240

[46] Martínez JM, Escudero C, Rodríguez N, Rubin S, Amils R. 2021. Subsurface and surface halophile communities of the chaotropic Salar de Uyuni. Environ Microbiol 23:3987–4001. doi:10.1111/1462-2920.15411. 33511754

[47] Mani K, Taib N, Hugoni M, Bronner G, Bragança JM, Debroas D. 2020. Transient dynamics of archaea and bacteria in sediments and brine across a salinity gradient in a solar saltern of Goa, India. Front Microbiol 11:1891. doi:10.3389/fmicb.2020.01891. 33013726 PMC7461921

[48] Oren A. 2015. Pyruvate: a key nutrient in hypersaline environments? Microorganisms 3:407–416. doi:10.3390/microorganisms3030407. 27682096 PMC5023246

[49] Konstantinidis KT, Rosselló-Móra R, Amann R. 2017. Uncultivated microbes in need of their own taxonomy. ISME J 11:2399–2406. doi:10.1038/ismej.2017.113. 28731467 PMC5649169

[50] Rosselló-Móra R, Amann R. 2015. Past and future species definitions for *Bacteria* and *Archaea*. Syst Appl Microbiol 38:209–216. doi:10.1016/j.syapm.2015.02.001. 25747618

[51] Rodríguez-Gijón A, Nuy JK, Mehrshad M, Buck M, Schulz F, Woyke T, Garcia SL. 2022. A genomic perspective across earth’s microbiomes reveals that genome size in Archaea and Bacteria is linked to ecosystem type and trophic strategy. Front Microbiol 12:1–9. doi:10.3389/fmicb.2021.761869. PMC8767057 35069467

[52] Kellner S, Spang A, Offre P, Szöllősi GJ, Petitjean C, Williams TA. 2018. Genome size evolution in the Archaea. Emerg Top Life Sci 2:595–605. doi:10.1042/ETLS20180021. 33525826 PMC7289037

[53] Grote J, Thrash JC, Huggett MJ, Landry ZC, Carini P, Giovannoni SJ, Rappé MS. 2012. Streamlining and core genome conservation among highly divergent members of the SAR11 clade. mBio 3:e00252–e00312. doi:10.1128/mBio.00252-12. 22991429 PMC3448164

[54] Klappenbach JA, Dunbar JM, Schmidt TM. 2000. rRNA operon copy number reflects ecological strategies of bacteria. Appl Environ Microbiol 66:1328–1333. doi:10.1128/AEM.66.4.1328-1333.2000. 10742207 PMC91988

[55] Hamm JN, Erdmann S, Eloe-Fadrosh EA, Angeloni A, Zhong L, Brownlee C, Williams TJ, Barton K, Carswell S, Smith MA, Brazendale S, Hancock AM, Allen MA, Raftery MJ, Cavicchioli R. 2019. Unexpected host dependency of antarctic Nanohaloarchaeota. Proc Natl Acad Sci USA 116:14661–14670. doi:10.1073/pnas.1905179116. 31253704 PMC6642349

[56] Giovannoni SJ, Thrash JC, Temperton B. 2014. Implications of streamlining theory for microbial ecology. ISME J 8:1553–1565. doi:10.1038/ismej.2014.60. 24739623 PMC4817614

[57] Dufresne A, Garczarek L, Partensky F. 2005. Accelerated evolution associated with genome reduction in a free-living prokaryote. Genome Biol 6:R14. doi:10.1186/gb-2005-6-2-r14. 15693943 PMC551534

[58] Lynch M, Conery JS. 2003. The origins of genome complexity. Science 302:1401–1404. doi:10.1126/science.1089370. 14631042

[59] Giovannoni SJ, Tripp HJ, Givan S, Podar M, Vergin KL, Baptista D, Bibbs L, Eads J, Richardson TH, Noordewier M, Rappé MS, Short JM, Carrington JC, Mathur EJ. 2005. Genetics: genome streamlining in a cosmopolitan oceanic bacterium. Science 309:1242–1245. doi:10.1126/science.1114057. 16109880

[60] Button DK, Robertson BR. 2001. Determination of DNA content of aquatic bacteria by flow cytometry. Appl Environ Microbiol 67:1636–1645. doi:10.1128/AEM.67.4.1636-1645.2001. 11282616 PMC92780

[61] Falb M, Müller K, Königsmaier L, Oberwinkler T, Horn P, von Gronau S, Gonzalez O, Pfeiffer F, Bornberg-Bauer E, Oesterhelt D. 2008. Metabolism of halophilic archaea. Extremophiles 12:177–196. doi:10.1007/s00792-008-0138-x. 18278431 PMC2262144

[62] Anderson I, Scheuner C, Göker M, Mavromatis K, Hooper SD, Porat I, Klenk H-P, Ivanova N, Kyrpides N. 2011. Novel insights into the diversity of catabolic metabolism from ten haloarchaeal genomes. PLoS One 6:e20237. doi:10.1371/journal.pone.0020237. 21633497 PMC3102087

[63] Verhees CH, Kengen SWM, Tuininga JE, Schut GJ, Adams MWW, de Vos WM, van der Oost J. 2003. The unique features of glycolytic pathways in Archaea. Biochem J 375:231–246. doi:10.1042/BJ20021472. 12921536 PMC1223704

[64] Sato T, Atomi H, Imanaka T. 2007. Archaeal type III RuBisCOs function in a pathway for AMP metabolism. Science 315:1003–1006. doi:10.1126/science.1135999. 17303759

[65] Schönheit P, Buckel W, Martin WF. 2016. On the origin of heterotrophy. Trends Microbiol 24:12–25. doi:10.1016/j.tim.2015.10.003. 26578093

[66] Aono R, Sato T, Yano A, Yoshida S, Nishitani Y, Miki K, Imanaka T, Atomi H. 2012. Enzymatic characterization of amp phosphorylase and ribose-1,5-bisphosphate isomerase functioning in an archaeal amp metabolic pathway. J Bacteriol 194:6847–6855. doi:10.1128/JB.01335-12. 23065974 PMC3510611

[67] Bulzu PA, Andrei AŞ, Salcher MM, Mehrshad M, Inoue K, Kandori H, Beja O, Ghai R, Banciu HL. 2019. Casting light on Asgardarchaeota metabolism in a sunlit microoxic niche. Nat Microbiol 4:1129–1137. doi:10.1038/s41564-019-0404-y. 30936485

[68] Castelle CJ, Banfield JF. 2018. Major new microbial groups expand diversity and alter our understanding of the tree of life. Cell 172:1181–1197. doi:10.1016/j.cell.2018.02.016. 29522741

[69] Sorokin D, Makarova KS, Abbas B, Ferrer M, Golyshin PN, Galinski EA, Ciordia S, Mena MC, Merkel AY, Wolf YI, Van Loosdrecht MCM, Koonin EV. 2017. Discovery of extremely halophilic, methyl-reducing euryarchaea provides insights into the evolutionary origin of methanogenesis. Nat Microbiol 2:17081. doi:10.1038/nmicrobiol.2017.81. 28555626 PMC5494993

[70] Sato T, Utashima S, Yoshii Y, Hirata K, Kanda S, Onoda Y, Qiang Jin J, Xiao S, Minami R, Fukushima H, Noguchi A, Manabe Y, Fukase K, Atomi H. 2022. A non-carboxylating pentose bisphosphate pathway in halophilic archaea. Commun Biol 5:1–13. doi:10.1038/s42003-022-04247-2. 36434094 PMC9700705

[71] Oren A. 2011. Thermodynamic limits to microbial life at high salt concentrations. Environ Microbiol 13:1908–1923. doi:10.1111/j.1462-2920.2010.02365.x. 21054738

[72] Gunde-Cimerman N, Oren A, Plemenitaš A. 2018. Strategies of adaptation of microorganisms of the three domains of life to high salt concentrations. FEMS Microbiol Rev 42:353–375. doi:10.1093/femsre/fuy009. 29529204

[73] Booth IR, Blount P. 2012. The MscS and MscL families of mechanosensitive channels act as microbial emergency release valves. J Bacteriol 194:4802–4809. doi:10.1128/JB.00576-12. 22685280 PMC3430326

[74] Oren A, Ventosa A, Grant WD. 1997. Proposed minimal standards for description of new taxa in the order *Halobacteriales*. Int J Syst Bacteriol 47:233–238. doi:10.1099/00207713-47-1-233.

[75] Oren A, Arahal DR, Ventosa A. 2009. Emended descriptions of genera of the family *Halobacteriaceae*. Int J Syst Evol Microbiol 59:637–642. doi:10.1099/ijs.0.008904-0. 19244452

[76] DeLong EF. 1992. Archaea in coastal marine environments. Proc Natl Acad Sci USA 89:5685–5689. doi:10.1073/pnas.89.12.5685. 1608980 PMC49357

[77] Arahal DR, Dewhirst FE, Paster BJ, Volcani BE, Ventosa A. 1996. Phylogenetic analyses of some extremely halophilic archaea isolated from Dead Sea water, determined on the basis of their 16S rRNA sequences. Appl Environ Microbiol 62:3779–3786. doi:10.1128/aem.62.10.3779-3786.1996. 8837434 PMC168186

[78] Bankevich A, Nurk S, Antipov D, Gurevich AA, Dvorkin M, Kulikov AS, Lesin VM, Nikolenko SI, Pham S, Prjibelski AD, Pyshkin AV, Sirotkin AV, Vyahhi N, Tesler G, Alekseyev MA, Pevzner PA. 2012. SPAdes: A new genome assembly algorithm and its applications to single-cell sequencing. J Comput Biol 19:455–477. doi:10.1089/cmb.2012.0021. 22506599 PMC3342519

[79] Parks DH, Imelfort M, Skennerton CT, Hugenholtz P, Tyson GW. 2015. CheckM: Assessing the quality of microbial genomes recovered from isolates, single cells, and metagenomes. Genome Res 25:1043–1055. doi:10.1101/gr.186072.114. 25977477 PMC4484387

[80] Gurevich A, Saveliev V, Vyahhi N, Tesler G. 2013. QUAST: quality assessment tool for genome assemblies. Bioinformatics 29:1072–1075. doi:10.1093/bioinformatics/btt086. 23422339 PMC3624806

[81] Hyatt D, Chen G-L, Locascio PF, Land ML, Larimer FW, Hauser LJ. 2010. Prodigal: prokaryotic gene recognition and translation initiation site identification. BMC Bioinformatics 11:119. doi:10.1186/1471-2105-11-119. 20211023 PMC2848648

[82] Seemann T. 2014. Prokka: rapid prokaryotic genome annotation. Bioinformatics 30:2068–2069. doi:10.1093/bioinformatics/btu153. 24642063

[83] Kanehisa M, Sato Y, Morishima K. 2016. BlastKOALA and GhostKOALA: KEGG tools for functional characterization of genome and metagenome sequences. J Mol Biol 428:726–731. doi:10.1016/j.jmb.2015.11.006. 26585406

[84] Kanehisa M, Sato Y, Kawashima M. 2022. KEGG mapping tools for uncovering hidden features in biological data. Protein Sci 31:47–53. doi:10.1002/pro.4172. 34423492 PMC8740838

[85] Rodriguez-R LM, Konstantinidis KT. 2016. The enveomics collection: a toolbox for specialized analyses of microbial genomes and metagenomes. PeerJ Preprints 4:e1900v1. doi:10.7287/peerj.preprints.1900v1.

[86] Rice P, Longden L, Bleasby A. 2000. EMBOSS: the European molecular biology open software suite. Trends Genet 16:276–277. doi:10.1016/s0168-9525(00)02024-2. 10827456

[87] Yoon SH, Ha SM, Kwon S, Lim J, Kim Y, Seo H, Chun J. 2017. Introducing EzBioCloud: a taxonomically united database of 16S rRNA gene sequences and whole-genome assemblies. Int J Syst Evol Microbiol 67:1613–1617. doi:10.1099/ijsem.0.001755. 28005526 PMC5563544

[88] Quast C, Pruesse E, Yilmaz P, Gerken J, Schweer T, Yarza P, Peplies J, Glöckner FO. 2013. The SILVA ribosomal RNA gene database project: improved data processing and web-based tools. Nucleic Acids Res 41:D590–D596. doi:10.1093/nar/gks1219. 23193283 PMC3531112

[89] Katoh K, Standley DM. 2013. MAFFT multiple sequence alignment software version 7: improvements in performance and usability. Mol Biol Evol 30:772–780. doi:10.1093/molbev/mst010. 23329690 PMC3603318

[90] Stamatakis A. 2014. RAxML version 8: a tool for phylogenetic analysis and post-analysis of large phylogenies. Bioinformatics 30:1312–1313. doi:10.1093/bioinformatics/btu033. 24451623 PMC3998144

[91] Edgar RC. 2004. MUSCLE: a multiple sequence alignment method with reduced time and space complexity. BMC Bioinformatics 5:113. doi:10.1186/1471-2105-5-113. 15318951 PMC517706

[92] Price MN, Dehal PS, Arkin AP. 2010. FastTree 2 – approximately maximum-likelihood trees for large alignments. PLoS One 5:e9490. doi:10.1371/journal.pone.0009490. 20224823 PMC2835736

[93] Letunic I, Bork P. 2021. Interactive Tree Of Life (iTOL) v5: an online tool for phylogenetic tree display and annotation. Nucleic Acids Res 49:W293–W296. doi:10.1093/nar/gkab301. 33885785 PMC8265157

[94] Gerhardt K, Ruiz-Perez CA, Rodriguez-R LM, Conrad RE, Konstantinidis KT. 2022. RecruitPlotEasy: an advanced read recruitment plot tool for assessing metagenomic population abundance and genetic diversity. Front Bioinform 1:1–5. doi:10.3389/fbinf.2021.826701. PMC9580866 36303791

[95] Lagkouvardos I, Joseph D, Kapfhammer M, Giritli S, Horn M, Haller D, Clavel T. 2016. IMNGS: a comprehensive open resource of processed 16S rRNA microbial profiles for ecology and diversity studies. Sci Rep 6:33721. doi:10.1038/srep33721. 27659943 PMC5034312

