# Supplementary material for: Discovery of the Streamlined Haloarchaeon Halorutilus salinus, Comprising a New Order Widespread in Hypersaline Environments across the World
Source: mSystems. 2023 Mar 21;8(2):e01198-22. doi: 10.1128/msystems.01198-22 (PMC10134839; doi:10.1128/msystems.01198-22)
Supplement: TABLE S4 [file msystems.01198-22-s0008.pdf]

	Accession number	Genome size (Mbp)	G+C (mol%)
Haladaptatus paucihalophilus DX253[T]	AQXI01000001	4.3	61.8
Halalkaliarchaeum desulfuricum AArc-Sl[T]	CP025066	3.3	63.1
Halanaeroarchaeum sulfurireducens HSR2[T]	CP008874	2.1	63.3
Halapricum salinum CBA1105[T]	BBMO01000001	3.5	63.7
Halarchaeum acidiphilum MH1-52-1[T]	BATA01000001	2.6	67.4
Halarchaeobius iranensis EB21[T]	FNIA01000001	3.8	67.3
Haloarcula vallismortis ATCC 29715[T]	AOLQ01000001	3.9	61.8
Halobacterium salinarum NRC-1[T]	VRYN01000001	2.0	67.9
Halobaculum gomorrense DSM 9297[T]	FQWV01000001	3.2	68.6
Halobellus clavatus CGMCC 1.10118[T]	FNPB01000001	3.8	64.1
Halobiforma haloterrestris DSM 13078[T]	FOKW01000001	4.5	65.4
Halocalculus aciditolerans JCM 19596[T]	BMPG01000001	3.4	66.9
Halocatena pleomorpha SPP-AMP-1[T]	RRCH01000001	3.7	57.1
Halococcoides cellulosivorans HArcel1[T]	CP028858	2.7	65.7
Halococcus morrhuae DSM 1307[T]	AOMC01000001	3.0	63.9
Halodesulfurarchaeum formicicum HSR6[T]	CP016804	2.1	63.6
Haloferax volcanii DS2[T]	CP001956	2.8	66.6
Halogenticoccus soli SYSU A9-0[T]	PEND01000001	4.6	66.9
Halogeometricum borinquense DSM 11551[T]	AOHT01000001	3.9	60.0
Haloglomus irregulare F16-60[T]	QMDX01000001	4.0	68.1
Halogranum rubrum CGMCC 1.7738[T]	FOTC01000001	4.6	62.2
Halolamina pelagica CGMCC 1.10329[T]	FOXI01000001	3.2	67.1
Halomarina oriensis JCM 16495[T]	WSZK01000001	4.1	66.8
Halomicrobium mukohatei DSM 12286[T]	CP001688	3.1	65.6
Halomicrococcus hydrotolerans H22[T]	QNGA01000001	5.6	63.0
Haloparvum sediment DSYS4[T]	LKIR01000001	3.2	68.3
Halopelagius inordinatus CGMCC 1.7739[T]	FOOQ01000001	3.5	65.0
Halopiger xanuadensis SH-6[T]	CP002839	3.7	66.0
Haloplanus natans DSM 17983[T]	ATYM01000001	3.8	65.0
Haloprofundus marisrubri SB9[T]	LOPU01000001	3.9	62.5
Haloquadratum walsbyi C23[T]	FR746099	3.1	47.8
Halorhabdus utahensis DSM 12940[T]	CP001687	3.1	62.9
Halorientalis regularis IBRC-M 10760	FNBK01000001	4.0	65.0

	Accession number	Genome size (Mbp)	G+C (mol%)
Halorubellus salinus GX3^T	JAJCVI010000001	4.1	68.0
Halorussus rarus TBN4^T	QPMJ01000001	4.4	68.0
Halosegnis rubeus F17-44^T	QJOW01000001	2.7	64.3
Halosiccatus urmianus IBRC-M 10911^T	CP084090	3.3	68.5
Halosimplex carlsbadense 2-9-1^T	AOIU01000001	4.7	67.7
Halostagnicola larsenii XH-48^T	CP007055	2.8	62.0
Halostella salina CBA1114^T	RCIH01000001	3.7	67.2
Haloterrigena turkmenica DSM 5511^T	CP001860	3.9	65.8
Halovenus aranensis IBRC-M 10015^T	FNFC01000001	3.3	61.1
Halovivax asiaticus JCM 14624^T	AOIQ01000002	3.2	64.5
Halopenitus persicus DC30^T	FNPC01000001	3.4	65.5
Halomicroarcula pellucida CECT 7537^T	RKLW00000000	3.9	65.5
Halonotius pteroides CECT 7525^T	QMDW00000000	3.0	59.5
Halorubrum saccharovorum DSM 1137^T	AOJE01000001	3.4	66.9
Natrarchaeobaculum sulfurireducens AArc1^T	CP024047	3.5	62.9
Natrarchaeobius chitinivorans AArcht4^T	REGA01000001	4.6	61.9
Natrialba asiatica DSM 12278^T	AOIO01000001	4.4	62.4
Natrinema pellirubrum DSM 15624^T	CP003372	3.8	64.9
Natronobacterium gregoryi SP2^T	CP003377	3.8	62.2
Natronococcus ocultus SP4^T	CP003929	4.0	64.9
Natronolimnobius baerhuensis JCM 12253^T	BBCV01000001	3.9	60.1
Natronolimnohabitans innermongolicus JCM 12255^T	AOHZ01000001	4.6	64.3
Natronomonas pharaonis DSM 2160^T	CR936257	2.6	63.4
Natronorubrum bangense JCM 10635^T	AOHY01000001	4.1	60.4
Salarchaeum japonicum JCM 16327^T	CP085324	2.2	68.0
Salinadaptatus halalkaliphilus XQ-INN 246^T	RBZW01000001	4.5	62.1
Salinibaculum litoreum HD8-45^T	CP046866	3.3	62.3
Salinigranum rubrum GX10^T	CP026309	4.3	66.9
Salinirubrum litoreum XD46^T	JAJCVJ010000001	4.1	66.9
Salinirussus salinus YGH44^T	WOWO01000001	3.4	68.7
Saliphagus infecundisoli YIM 93745^T	JAIWHV010000001	4.5	64.2
