# Supplementary figures and images for: Discovery of the Streamlined Haloarchaeon Halorutilus salinus, Comprising a New Order Widespread in Hypersaline Environments across the World
Source: mSystems. 2023 Mar 21;8(2):e01198-22. doi: 10.1128/msystems.01198-22 (PMC10134839; doi:10.1128/msystems.01198-22)

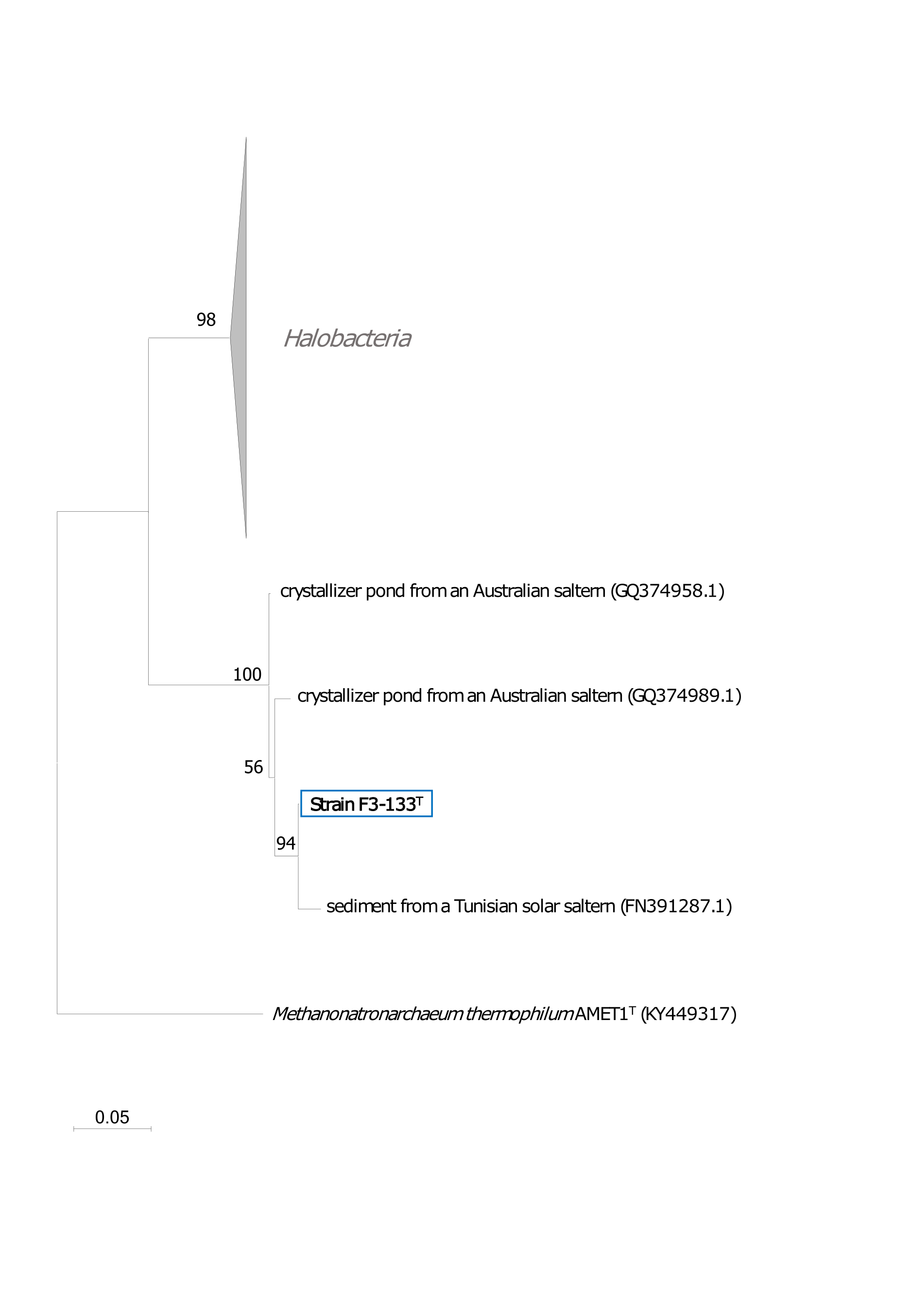

Supplement: FIG S1 [file msystems.01198-22-s0001.tif]

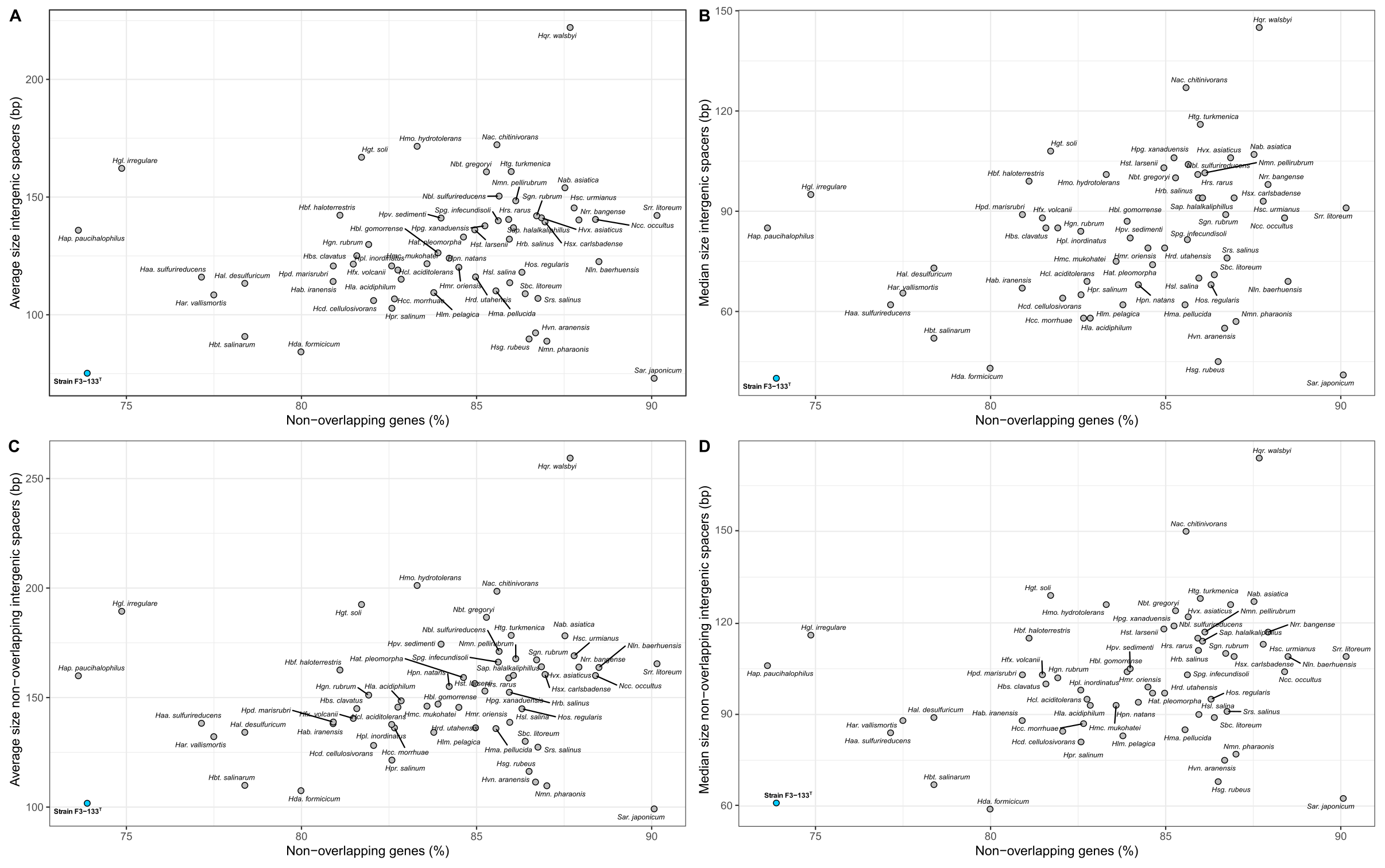

Supplement: FIG S2 [file msystems.01198-22-s0002.tif]

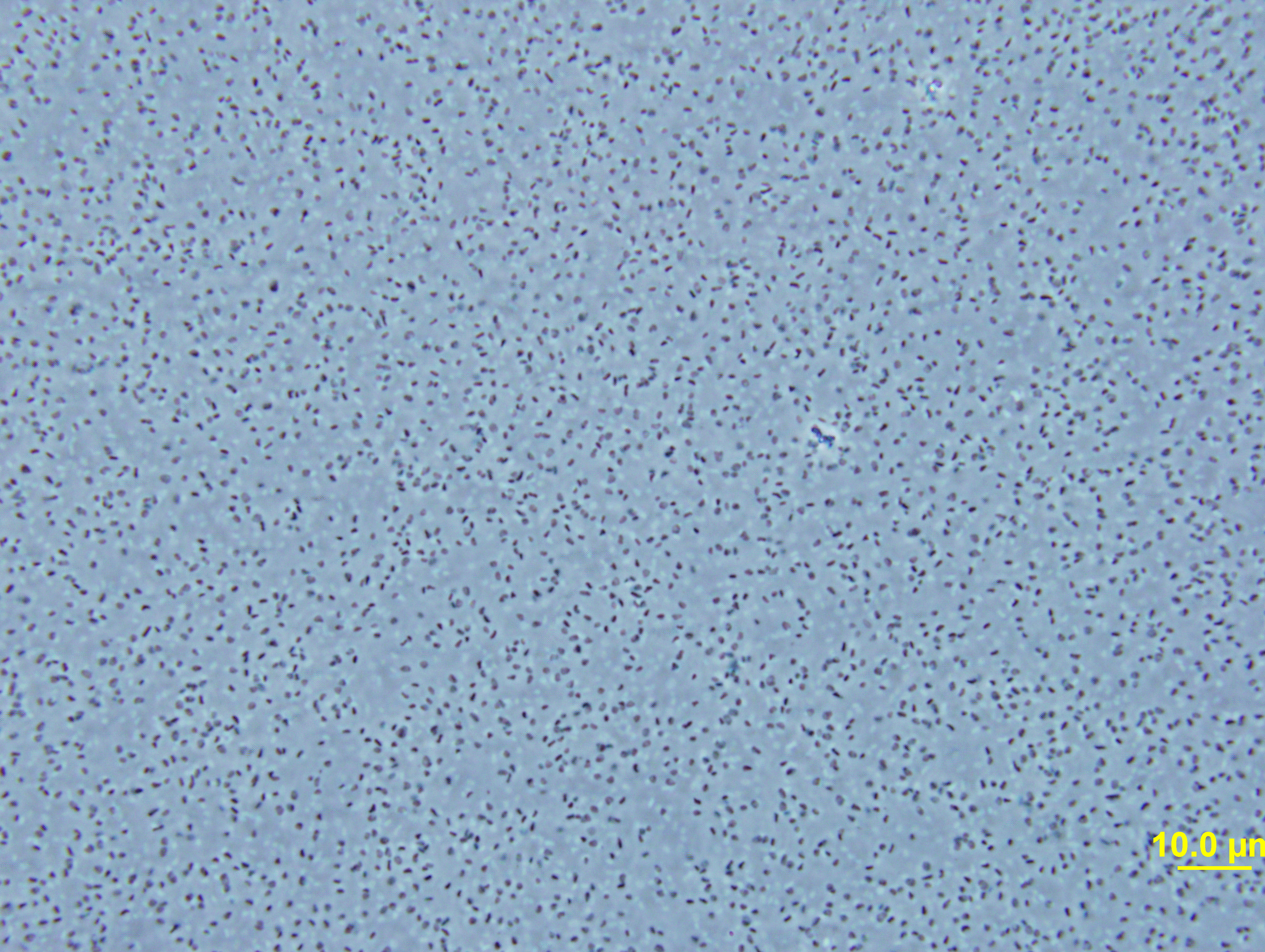

Supplement: FIG S3 [file msystems.01198-22-s0003.tif]

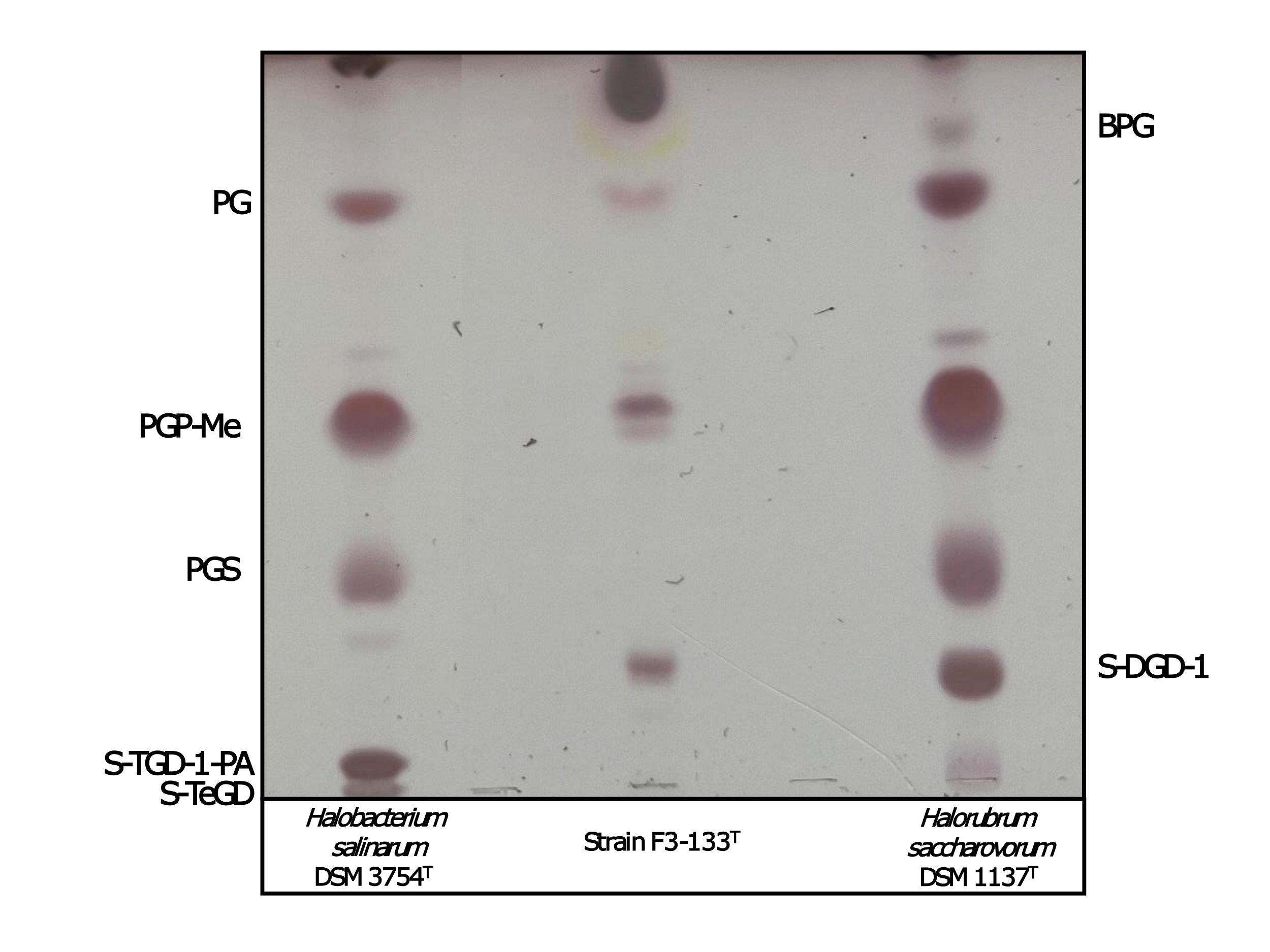

Supplement: FIG S4 [file msystems.01198-22-s0004.tif]
