# Supplementary material for: Discovery of the Streamlined Haloarchaeon Halorutilus salinus, Comprising a New Order Widespread in Hypersaline Environments across the World
Source: mSystems. 2023 Mar 21;8(2):e01198-22. doi: 10.1128/msystems.01198-22 (PMC10134839; doi:10.1128/msystems.01198-22)
Supplement: TABLE S1 [file msystems.01198-22-s0005.pdf]

Database	Accession number	Environment	Geographic location	Depth	Salinity (%)	pH	Reference
Amplicon	SRR1560574	Meromictic hypersaline lake	Fara Fund Lake (Romania)	3m	29	6.1	(1)
Amplicon	SRR1560593	Meromictic hypersaline lake	Fara Fund Lake (Romania)	11m	33	5.9	(1)
Amplicon	ERR580980	Hypersaline lake sediment	Lake Strawbridge (Australia)	0-1 cm	18.6	8.3	(2)
Amplicon	ERR580981	Hypersaline lake sediment	Lake Strawbridge (Australia)	1-10 cm	11.8	8.2	(2)
Amplicon	ERR580982	Hypersaline lake sediment	Lake Strawbridge (Australia)	>10 cm	5.1	8.1	(2)
Amplicon	ERR580977	Hypersaline lake sediment	Lake Strawbridge (Australia)	0-1 cm	18.6	8.3	(2)
Amplicon	ERR580978	Hypersaline lake sediment	Lake Strawbridge (Australia)	1-10	11.8	8.2	(2)
Amplicon	SRR2976523	Stromatolite	Great Salt Lake (USA)	-	12-32	7.2-7.9	(3)
Clone library	GQ374958	Saltern	Bajool (Australia)	25 cm	34	7.2	(4)
Clone library	GQ374989	Saltern	Bajool (Australia)	25 cm	34	7.2	(4)
Clone library	FN391287	Saltern sediment	Sfax (Tunisia)	2-5 cm	25-30	-	(5)

1. Andrei A-Ş, Robeson MS, Baricz A, Coman C, Muntean V, Ionescu A, Etiope G, Alexe M, Sicora CI, Podar M, Banciu HL. 2015. Contrasting taxonomic stratification of microbial communities in two hypersaline meromictic lakes. ISME J 9:2642–2656.

2. Weigold P, Ruecker A, Loesekann-Behrens T, Kappler A, Behrens S. 2016. Ribosomal tag pyrosequencing of DNA and RNA reveals “rare” taxa with high protein synthesis potential in the sediment of a hypersaline lake in western Australia. Geomicrobiol J 33:426–440.

3. Lindsay MR, Anderson C, Fox N, Scofield G, Allen J, Anderson E, Bueter L, Poudel S, Sutherland K, Munson-McGee JH, Van Nostrand JD, Zhou J, Spear JR, Baxter BK, Lageson DR, Boyd ES. 2017. Microbialite response to an anthropogenic salinity gradient in Great Salt Lake, Utah. Geobiology 15:131–145.

4. Oh D, Porter K, Russ B, Burns D, Dyall-Smith M. 2010. Diversity of *Haloquadratum* and other haloarchaea in three, geographically distant, Australian saltern crystallizer ponds. Extremophiles 14:161–169.

5. Baati H, Guermazi S, Gharsallah N, Sghir A, Ammar E. 2010. Novel prokaryotic diversity in sediments of Tunisian multipond solar saltern. Res Microbiol 161:573–582.
