# Supplementary material for: Discovery of the Streamlined Haloarchaeon Halorutilus salinus, Comprising a New Order Widespread in Hypersaline Environments across the World
Source: mSystems. 2023 Mar 21;8(2):e01198-22. doi: 10.1128/msystems.01198-22 (PMC10134839; doi:10.1128/msystems.01198-22)
Supplement: TABLE S2 [file msystems.01198-22-s0006.pdf]

Database accession number	Environment	Geographic location
ERR833309	Salt lagoon	Punta Cormorant (Ecuador)
ERR833310	Salt lagoon	Punta Cormorant (Ecuador)
ERR833369	Hypersaline lake	Lake Tyrrel (Australia)
ERR833370	Hypersaline lake	Lake Tyrrel (Australia)
ERR833371	Hypersaline lake	Lake Tyrrel (Australia)
ERR833372	Hypersaline lake	Lake Tyrrel (Australia)
ERR833373	Hypersaline lake	Lake Tyrrel (Australia)
ERR833374	Hypersaline lake	Lake Tyrrel (Australia)
ERR833376	Solar saltern	Cheetam (Australia)
ERR833377	Solar saltern	Cheetam (Australia)
ERR833378	Solar saltern	Cheetam (Australia)
ERR833379	Solar saltern	Cheetam (Australia)
ERR833380	Solar saltern	Cheetam (Australia)
ERR833381	Solar saltern	Cheetam (Australia)
ERR833382	Solar saltern	Cheetam (Australia)
ERR833383	Solar saltern	Cheetam (Australia)
ERR833525	Salt pond	Isla Carmen (Mexico)
ERR3624103	Hypersaline lake sediment	Lake Strawbridge (Australia)
ERR3624104	Hypersaline lake sediment	Lake Strawbridge (Australia)
ERR3624105	Hypersaline lake sediment	Lake Strawbridge (Australia)
ERR3624502	Hypersaline lake sediment	Lake Strawbridge (Australia)
SRR12122875	Microbial mat	Tebenquiche lagoon (Chile)
SRR12122876	Microbial mat	Tebenquiche lagoon (Chile)
ERR5388654	Solar saltern	Es Trenc (Spain)
ERR5414699	Solar saltern sediment	S´Avall (Spain)
ERR5414701	Solar saltern sediment	S´Avall (Spain)
ERR5414649	Solar saltern sediment	S´Avall (Spain)
ERR5414700	Solar saltern sediment	S´Avall (Spain)
ERR1739731	Hypersaline lake	Lake Meyghan (Iran)
ERR1739732	Hypersaline lake	Lake Meyghan (Iran)
ERR1739733	Hypersaline lake	Lake Meyghan (Iran)
ERR1742997	Hypersaline lake	Lake Meyghan (Iran)
ERR1742998	Hypersaline lake	Lake Meyghan (Iran)
ERR1742999	Hypersaline lake	Lake Meyghan (Iran)
ERR1743000	Hypersaline lake	Lake Meyghan (Iran)
ERR1743001	Hypersaline lake	Lake Meyghan (Iran)
ERR1743002	Hypersaline lake	Lake Meyghan (Iran)
SRR5637210	Hypersaline lake	Lake Tyrrell (Australia)
SRR5637211	Hypersaline lake	Lake Tyrrell (Australia)
SRR4027687	Hypersaline lake	Deep Lake (Antarctica)
SRR4027688	Hypersaline lake	Deep Lake (Antarctica)
SRR6963308	Hypersaline lake sediment	Salton Sea (USA)
SRR6963311	Hypersaline lake sediment	Salton Sea (USA)
SRR6963312	Hypersaline lake sediment	Salton Sea (USA)
SRR6963352	Hypersaline lake sediment	Salton Sea (USA)
SRR6963479	Hypersaline lake sediment	Salton Sea (USA)
SRR6963480	Hypersaline lake sediment	Salton Sea (USA)

Database accession number	Environment	Geographic location
SRR6963491	Hypersaline lake sediment	Salton Sea (USA)
SRR6963492	Hypersaline lake sediment	Salton Sea (USA)
SRR6963587	Hypersaline lake sediment	Salton Sea (USA)
SRR6963591	Hypersaline lake sediment	Salton Sea (USA)
SRR988245	Solar saltern	Isla Cristina (Spain)
PRJNA890281	Solar saltern	Isla Cristina (Spain)
SRR944625	Solar saltern	Santa Pola (Spain)
SRR979792	Solar saltern	Santa Pola (Spain)
SRR13628065	Cold saline spring	Axel Heiberg Island (Canada)
SRR13628066	Cold saline spring	Axel Heiberg Island (Canada)
ERR5395817	Solar saltern	Es Trenc (Spain)
ERR5396066	Solar saltern	Es Trenc (Spain)
ERR2688510	Solar saltern	Es Trenc (Spain)
ERR2688511	Solar saltern	Es Trenc (Spain)
ERR2688512	Solar saltern	Es Trenc (Spain)
ERR2688513	Solar saltern	Es Trenc (Spain)
ERR2688514	Solar saltern	Es Trenc (Spain)
ERR2688515	Solar saltern	Es Trenc (Spain)
ERR2688516	Solar saltern	Es Trenc (Spain)
ERR2688517	Solar saltern	Es Trenc (Spain)
ERR2688518	Solar saltern	Es Trenc (Spain)
ERR2688519	Solar saltern	Es Trenc (Spain)
ERR2688520	Solar saltern	Es Trenc (Spain)
ERR5411942	Solar saltern	Es Trenc (Spain)
ERR5414394	Solar saltern	Es Trenc (Spain)
ERR5414395	Solar saltern	Es Trenc (Spain)
ERR5414396	Solar saltern	Es Trenc (Spain)
ERR5415056	Solar saltern	Es Trenc (Spain)
ERR5415062	Solar saltern	Es Trenc (Spain)
ERR5002016	Salt marsh soil	Odiel saltmarshes (Spain)
ERR5002017	Salt marsh soil	Odiel saltmarshes (Spain)
ERR5002018	Salt marsh soil	Odiel saltmarshes (Spain)
ERR5002019	Salt marsh soil	Odiel saltmarshes (Spain)
ERR5002020	Salt marsh soil	Odiel saltmarshes (Spain)
ERR5002021	Salt marsh soil	Odiel saltmarshes (Spain)
ERR5002022	Salt marsh soil	Odiel saltmarshes (Spain)
ERR5002023	Salt marsh soil	Odiel saltmarshes (Spain)
ERR5002024	Salt marsh soil	Odiel saltmarshes (Spain)
ERR5002025	Salt marsh soil	Odiel saltmarshes (Spain)
ERR5002026	Salt marsh soil	Odiel saltmarshes (Spain)
ERR5003295	Salt marsh soil	Odiel saltmarshes (Spain)
ERR5003296	Salt marsh soil	Odiel saltmarshes (Spain)
ERR5003297	Salt marsh soil	Odiel saltmarshes (Spain)
ERR5003298	Salt marsh soil	Odiel saltmarshes (Spain)
ERR5003299	Salt marsh soil	Odiel saltmarshes (Spain)
ERR5003300	Salt marsh soil	Odiel saltmarshes (Spain)
ERR5003301	Salt marsh soil	Odiel saltmarshes (Spain)

Database accession number	Environment	Geographic location
ERR5003302	Salt marsh soil	Odiel saltmarshes (Spain)
ERR5003303	Salt marsh soil	Odiel saltmarshes (Spain)
ERR5003304	Salt marsh soil	Odiel saltmarshes (Spain)
ERR5003305	Salt marsh soil	Odiel saltmarshes (Spain)
ERR5003997	Salt marsh soil	Odiel saltmarshes (Spain)
ERR5003998	Salt marsh soil	Odiel saltmarshes (Spain)
ERR5003999	Salt marsh soil	Odiel saltmarshes (Spain)
ERR5004000	Salt marsh soil	Odiel saltmarshes (Spain)
ERR5004001	Salt marsh soil	Odiel saltmarshes (Spain)
ERR5004002	Salt marsh soil	Odiel saltmarshes (Spain)
ERR5004003	Salt marsh soil	Odiel saltmarshes (Spain)
ERR5004004	Salt marsh soil	Odiel saltmarshes (Spain)
ERR5004005	Salt marsh soil	Odiel saltmarshes (Spain)
ERR5004006	Salt marsh soil	Odiel saltmarshes (Spain)
ERR5004007	Salt marsh soil	Odiel saltmarshes (Spain)
ERR5004008	Salt marsh soil	Odiel saltmarshes (Spain)
ERR5004009	Salt marsh soil	Odiel saltmarshes (Spain)
ERR5004010	Salt marsh soil	Odiel saltmarshes (Spain)
ERR5004011	Salt marsh soil	Odiel saltmarshes (Spain)
ERR5004012	Salt marsh soil	Odiel saltmarshes (Spain)
ERR5004013	Salt marsh soil	Odiel saltmarshes (Spain)
ERR5004014	Salt marsh soil	Odiel saltmarshes (Spain)
ERR5004015	Salt marsh soil	Odiel saltmarshes (Spain)
ERR5004016	Salt marsh soil	Odiel saltmarshes (Spain)
ERR5004017	Salt marsh soil	Odiel saltmarshes (Spain)
ERR5004018	Salt marsh soil	Odiel saltmarshes (Spain)
ERR5004019	Salt marsh soil	Odiel saltmarshes (Spain)
ERR5004020	Salt marsh soil	Odiel saltmarshes (Spain)
ERR5004784	Salt marsh soil	Odiel saltmarshes (Spain)
ERR5004785	Salt marsh soil	Odiel saltmarshes (Spain)
ERR5004786	Salt marsh soil	Odiel saltmarshes (Spain)
ERR5004787	Salt marsh soil	Odiel saltmarshes (Spain)
ERR5004788	Salt marsh soil	Odiel saltmarshes (Spain)
ERR5004789	Salt marsh soil	Odiel saltmarshes (Spain)
ERR5004790	Salt marsh soil	Odiel saltmarshes (Spain)
ERR5004791	Salt marsh soil	Odiel saltmarshes (Spain)
ERR5004792	Salt marsh soil	Odiel saltmarshes (Spain)
ERR5004793	Salt marsh soil	Odiel saltmarshes (Spain)
ERR5004794	Salt marsh soil	Odiel saltmarshes (Spain)
ERR5004795	Salt marsh soil	Odiel saltmarshes (Spain)
ERR5004796	Salt marsh soil	Odiel saltmarshes (Spain)
ERR5004797	Salt marsh soil	Odiel saltmarshes (Spain)
ERR5004798	Salt marsh soil	Odiel saltmarshes (Spain)
ERR5004799	Salt marsh soil	Odiel saltmarshes (Spain)
ERR5004800	Salt marsh soil	Odiel saltmarshes (Spain)
ERR5004801	Salt marsh soil	Odiel saltmarshes (Spain)
ERR5004802	Salt marsh soil	Odiel saltmarshes (Spain)

Database accession number	Environment	Geographic location
ERR5004803	Salt marsh soil	Odiel saltmarshes (Spain)
ERR5004804	Salt marsh soil	Odiel saltmarshes (Spain)
ERR5004805	Salt marsh soil	Odiel saltmarshes (Spain)
ERR5004806	Salt marsh soil	Odiel saltmarshes (Spain)
ERR5004807	Salt marsh soil	Odiel saltmarshes (Spain)
SRR12551239	Hypersaline soil from saltern	Cabo Frio (Brazil)
SRR12551240	Hypersaline soil from saltern	Cabo Frio (Brazil)
SRR12551241	Hypersaline soil from saltern	Cabo Frio (Brazil)
SRR12551242	Hypersaline soil from saltern	Cabo Frio (Brazil)
SRR12551243	Hypersaline soil from saltern	Cabo Frio (Brazil)
SRR12551244	Hypersaline soil from saltern	Cabo Frio (Brazil)
SRR10083178	Solar saltern	Ribandar (India)
ERR2683605	Solar saltern	Es Trenc (Spain)
ERR2683606	Solar saltern	Es Trenc (Spain)
ERR2683607	Solar saltern	Es Trenc (Spain)
ERR2683608	Solar saltern	Es Trenc (Spain)
ERR2683609	Solar saltern	Es Trenc (Spain)
ERR2683610	Solar saltern	Es Trenc (Spain)
ERR2683611	Solar saltern	Es Trenc (Spain)
ERR2683612	Solar saltern	Es Trenc (Spain)
ERR2683613	Solar saltern	Es Trenc (Spain)
ERR2683614	Solar saltern	Es Trenc (Spain)
ERR2683615	Solar saltern	Es Trenc (Spain)
ERR2683616	Solar saltern	Es Trenc (Spain)
ERR2683617	Solar saltern	Es Trenc (Spain)
ERR2683618	Solar saltern	Es Trenc (Spain)
ERR2683619	Solar saltern	Es Trenc (Spain)
ERR2683620	Solar saltern	Es Trenc (Spain)
ERR2683621	Solar saltern	Es Trenc (Spain)
ERR2683622	Solar saltern	Es Trenc (Spain)
ERR2683623	Solar saltern	Es Trenc (Spain)
ERR2683624	Solar saltern	Es Trenc (Spain)
ERR2683625	Solar saltern	Es Trenc (Spain)
ERR2683626	Solar saltern	Es Trenc (Spain)
ERR2683627	Solar saltern	Es Trenc (Spain)
ERR2683628	Solar saltern	Es Trenc (Spain)
ERR2683629	Solar saltern	Es Trenc (Spain)
ERR2683630	Solar saltern	Es Trenc (Spain)
ERR2683631	Solar saltern	Es Trenc (Spain)
ERR2683632	Solar saltern	Es Trenc (Spain)
ERR2683633	Solar saltern	Es Trenc (Spain)
ERR2683634	Solar saltern	Es Trenc (Spain)
ERR2683635	Solar saltern	Es Trenc (Spain)
ERR2683636	Solar saltern	Es Trenc (Spain)
ERR2683637	Solar saltern	Es Trenc (Spain)
ERR2683638	Solar saltern	Es Trenc (Spain)
ERR2683639	Solar saltern	Es Trenc (Spain)

Database accession number	Environment	Geographic location
ERR2683640	Solar saltern	Es Trenc (Spain)
ERR2683641	Solar saltern	Es Trenc (Spain)
ERR2683642	Solar saltern	Es Trenc (Spain)
ERR2683643	Solar saltern	Es Trenc (Spain)
ERR2683644	Solar saltern	Es Trenc (Spain)
ERR2683645	Solar saltern	Es Trenc (Spain)
ERR5981341	Solar saltern	Velddrif (South Africa)
ERR5979340	Solar saltern	S´Avall (Spain)
ERR5981342	Hypersaline lake	Great Salt Lake (USA)
ERR5988144	Solar saltern sediment	S´Avall (Spain)
ERR5988145	Solar saltern sediment	S´Avall (Spain)
ERR5988146	Solar saltern sediment	S´Avall (Spain)
SRR18572986	Hypersaline lake	Qijiaojing Lake (China)
SRR18572987	Hypersaline lake	Qijiaojing Lake (China)
SRR18572988	Hypersaline lake	Qijiaojing Lake (China)
SRR18572989	Hypersaline lake	Qijiaojing Lake (China)
SRR18572990	Hypersaline lake	Qijiaojing Lake (China)
SRR18572991	Hypersaline lake	Qijiaojing Lake (China)
SRR18572992	Hypersaline lake	Qijiaojing Lake (China)
SRR18572993	Hypersaline lake	Qijiaojing Lake (China)
SRR18572994	Hypersaline lake	Qijiaojing Lake (China)
SRR17687544	Solar saltern	Wendeng (China)
SRR17687545	Solar saltern	Wendeng (China)
SRR17687546	Solar saltern	Wendeng (China)
SRR17687547	Solar saltern	Wendeng (China)
SRR17687548	Solar saltern	Wendeng (China)
SRR17687549	Solar saltern	Wendeng (China)
SRR17687550	Solar saltern	Wendeng (China)
SRR17687551	Solar saltern	Wendeng (China)
SRR17687552	Solar saltern	Wendeng (China)
SRR17687553	Solar saltern	Wendeng (China)
SRR17687554	Solar saltern	Wendeng (China)
SRR17687555	Solar saltern	Wendeng (China)
SRR17687556	Solar saltern	Wendeng (China)
SRR17687557	Solar saltern	Wendeng (China)
SRR17687558	Solar saltern	Wendeng (China)
SRR316684	Solar saltern	Santa Pola (Spain)
SRR1043601	Solar saltern	Santa Pola (Spain)
SRR1043669	Solar saltern	Santa Pola (Spain)
SRR1043670	Solar saltern	Santa Pola (Spain)
SRR1043606	Solar saltern	Santa Pola (Spain)
SRR1043668	Solar saltern	Santa Pola (Spain)
SRR328982	Solar saltern	Santa Pola (Spain)
SRR328983	Solar saltern	Santa Pola (Spain)
SRR062267	Solar saltern	Santa Pola (Spain)
SRR13926767	Solar saltern	Santa Pola (Spain)
SRR13926768	Solar saltern	Santa Pola (Spain)

Database accession number	Environment	Geographic location
SRR13926769	Solar saltern	Santa Pola (Spain)
SRR13926770	Solar saltern	Santa Pola (Spain)
