# Supplementary material for: Discovery of the Streamlined Haloarchaeon Halorutilus salinus, Comprising a New Order Widespread in Hypersaline Environments across the World
Source: mSystems. 2023 Mar 21;8(2):e01198-22. doi: 10.1128/msystems.01198-22 (PMC10134839; doi:10.1128/msystems.01198-22)
Supplement: TABLE S3 [file msystems.01198-22-s0007.pdf]

	$F3\text{-}133^T$
**Number of contigs**	17
**Total length (Mb)**	2.1
**Completeness (%)**	97.1
**Number of coding sequences**	2323
**GC content (mol%)**	59.8
**N50 (bp)**	189022
**rRNAs**	3
**tRNAs**	44
**Accession number**	RKLV00000000
