# Supplementary material for: Discovery of the Streamlined Haloarchaeon Halorutilus salinus, Comprising a New Order Widespread in Hypersaline Environments across the World
Source: mSystems. 2023 Mar 21;8(2):e01198-22. doi: 10.1128/msystems.01198-22 (PMC10134839; doi:10.1128/msystems.01198-22)
Supplement: TABLE S5 [file msystems.01198-22-s0009.pdf]

Characteristic	Strain F3-133^T
Cell size (µm)	1.1-1.4 x 0.4-0.7 µm
Colony pigmentation	Red
Colony size	1.5 mm
NaCl requirement (%) (w/v)	10-30 (optimum 25)
pH requirement	6-9 (optimum 7-7.5)
Temperature requirement (°C)	30-45 (optimum 37)
Indole production	-
H_2S production	-
Hydrolysis of:	
Gelatin	+
DNA	-
Aesculin	-
Tween 80	+
Production of acids from carbohydrates	
Positive for:	D,L-arabinose, D-cellobiose, L-citrulline, D-fructose, D-ribose and D-xylose
Negative for:	D-amygdalin, arbutin, dulcitol, D-galactose, D-glucose, glycerol, lactose, D-maltose, D-mannitol, D-mannose, D-melezitose, D-raffinose, D-saccharose, sorbitol, D-trehalose and L-xylitol
